# Temporal trends in the starting of insulin therapy in type 2 diabetes in Italy: data from the AMD Annals initiative

**DOI:** 10.1007/s40618-024-02306-5

**Published:** 2024-03-05

**Authors:** A. Giandalia, A. Nicolucci, M. Modugno, G. Lucisano, M. C. Rossi, V. Manicardi, A. Rocca, G. Di Cianni, P. Di Bartolo, R. Candido, D. Cucinotta, G. T. Russo

**Affiliations:** 1 Department of Clinical and Experimental Medicine, University of Messina, Via C. Valeria, 98100 Messina, Italy; 2 Center for Outcomes Research and Clinical Epidemiology, CORESEARCH, Pescara, Italy; 3 ASLBA-DSS10 Poliambulatorio, Triggiano, BA Italy; 4 Fondazione AMD, Rome, Italy; 5 SS Diabetes and Metabolic disease, Bassini Hospital Cinisello Balsamo, Milan, Italy; 6 Diabetes and Metabolic Diseases Unit, Livorno Hospital, Livorno, Italy; 7 Diabetes Unit, Local Healthcare Authority of Romagna, Ravenna, Italy; 8 Azienda Sanitaria Universitaria Giuliano Isontina, University of Trieste, Trieste, Italy

**Keywords:** Clinical inertia, Insulin therapy, Observational study, Type 2 diabetes, AMD Annals Initiative

## Abstract

**Aims:**

Opportunities and needs for starting insulin therapy in Type 2 diabetes (T2D) have changed overtime. We evaluated clinical characteristics of T2D subjects undergoing the first insulin prescription during a 15-year-observation period in the large cohort of the AMD Annals Initiative in Italy.

**Methods:**

Data on clinical and laboratory variables, complications and concomitant therapies and the effects on glucose control after 12 months were evaluated in T2D patients starting basal insulin as add-on to oral/non-insulin injectable agents, and in those starting fast-acting in add-on to basal insulin therapy in three 5-year periods (2005–2019).

**Results:**

We evaluated data from 171.688 T2D subjects who intensified therapy with basal insulin and 137.225 T2D patients who started fast-acting insulin. Overall, intensification with insulin occurred progressively earlier over time in subjects with shorter disease duration. Moreover, the percentage of subjects with HbA1c levels > 8% at the time of basal insulin initiation progressively decreased. The same trend was observed for fast-acting formulations. Clinical characteristics of subjects starting insulin did not change in the three study-periods, although all major risk factors improved overtime. After 12 months from the starting of basal or fast-acting insulin therapy, mean HbA1c levels decreased in all the three investigated time-periods, although mean HbA1c levels remained above the recommended target.

**Conclusions:**

In this large cohort of T2D subjects, a progressively earlier start of insulin treatment was observed during a long observation period, suggesting a more proactive prescriptive approach. However, after 12 months from insulin prescription, in many patients, HbA1c levels were still out-of-target.

**Supplementary Information:**

The online version contains supplementary material available at 10.1007/s40618-024-02306-5.

## Introduction

The prevalence of type 2 diabetes (T2D) is rising in many places in the world, including Italy, where it increased from 4.1 to 5.9% in the latest 20 years [1, 2]. This figure places an enormous burden on healthcare systems, payers, and providers as well as at individual and social level.

To contain this dangerous wave, national and international guidelines are regularly updated to include better therapeutic options to cope with the evolving evidence, with the major aim of preventing T2D chronic complications while maintaining an adequate quality of life [3].

Metformin is recommended as the first-line pharmacological treatment, while innovative drugs are indicated for cardio-renal protection [3–5]. Injectable therapies including insulin are well-established agents for T2D, although the indication on the appropriate timing for starting insulin treatment in the therapeutic algorithm varied overtime [4–6]. Moreover, the advantages and the cost-benefits of insulin analogs and their combination with new glucose-lowering drugs are still a matter of debate [7].

In spite of the availability of new, highly effective treatments for T2D care, there is still a large gap between the guidelines recommendations and the outcomes achieved in clinical practice. Thus, HbA1c levels are higher than those recommended in many T2D patients: in 2022, in Italy about 40% of all T2DM subjects in care at diabetes clinics had HbA1c levels > 7%, and about 20% of them had HbA1c levels > 8% [8] and this is commonly observed also in other cohorts [9–12].

Reasons behind the lack of glucose control achievement in about half of T2D patients worldwide is a complex issue involving several aspects. The first of them is related to the progressive nature of the disease, characterized by a continuous decline of beta-cell function [13, 14]. Consequently, insulin supplementation is often needed to attain good glucose control in T2D [15].

Notably, a substantial proportion of patients with suboptimal control often experience delays in treatment intensification, a phenomenon that has been termed clinical inertia, with profound negative impact on the onset and the progression of both micro- and macrovascular complications [16, 17].

It has been calculated that a 1-year delay in intensification of glucose-lowering treatment is associated with a 62% increased risk of cardiovascular events [17].

At this regard, a recent systematic review showed that clinical inertia involves over 50% of T2D patients, ranging from 35.4 to 85.8% in the USA, to over 60% in Canada, Brazil, and Thailand [18]. Consequently, a significant number of T2D patients would suffer poor glycemic control for quite a long time before treatment intensification with oral glucose-lowering drugs (OHA) or insulin.

On the other hand, clinical inertia is a complex and multifaceted phenomenon, not only concerning glycemic control. Notably, the objectives of T2D management also include the timely treatment of all cardiovascular risk factors and body weight management. Weight loss, in overweight or obese subjects with T2D, represents a fundamental and achievable goal with various therapeutic options, including drugs and a personalized approach [19]. Despite this, the burden of obesity is increasing among individuals with both T2D and T1D [20].

In Italy, a continuous initiative to monitoring diabetes care has been in place since 2006 by a network of diabetes clinics operating within the national healthcare system as a key strategy to overcome clinical inertia [21–23].

This initiative, promoted by the Italian Association of Medical Diabetologists (AMD), which allows the yearly evaluation and critical revision of patterns of diabetes care, has documented a clear improvement in T2D care overtime [8], with a tangible impact on clinical outcomes and related healthcare costs [24].

As part of this initiative, the aim of our study was to evaluate temporal trends in insulin prescription and patterns of use over time in the large population of T2D outpatients participating to the AMD Annals Initiative. In particular, in three quinquennia spanning a 15-year observation period, we evaluated potential changes in the clinical characteristics of subjects receiving the first prescription of basal and/or fast-acting insulin therapy, and its consequences on 12-month glucose control.

## Materials and methods

### The AMD annals initiative

This observational, longitudinal, retrospective study was conducted in a network of Italian diabetes clinics participating in the continuous quality improvement initiative called AMD Annals, promoted since 2006, by the Italian Association of Clinical Diabetologists (Associazione Medici Diabetologi, AMD) [8, 24]. A total of 258 centres, accounting for approximately one-third of all the clinics operating within the Italian National Healthcare System, share the same software for data extraction from electronic medical records. Data from participating centres are annually collected in a standardized format (AMD Data File) and centrally analysed. Notably, both patients and centers are anonymously extracted and analyzed. The database is anonymous and each patient is assigned a unique numerical code, allowing the longitudinal analysis of the same patient in different years. As requested by the Regulatory authority in Italy, due to the nature of the study, the AMD Initiative received an ethics approval. The project is conducted without allocation of extra resources or financial incentives.

For the purposes of the present study, data on all patients with T2D attending the participating diabetes clinics between 2005 and 2019 were used. Two different groups were identified: patients starting basal insulin and patients starting fast acting insulin as add on to ongoing basal insulin treatment, with or without oral agents.

### Statistical analyses

The index date for each patient was represented by the date of basal insulin or fast acting insulin start. Baseline characteristics at index date are reported separately for three 5-year periods (2005–2009; 2010–2014; 2015–2019) and treatment (start of basal insulin or fast acting insulin).

Baseline descriptive variables included age, sex, body weight, BMI, HbA1c, blood pressure, lipid profile, estimated glomerular filtration rate (eGFR; Chronic Kidney Disease Epidemiology Collaboration, CKD-EPI formula), and diabetes treatment.

In each cohort, HbA1c values were analysed at the index date and after 12 months.

Patients’ characteristics overall and by cohort were summarized as mean and standard deviation (continuous variables) or frequencies and percentages (categorical variables).

All statistical analyses were performed with SAS software, version 9.4 (SAS Institute Inc., Cary, NC).

## Results

### Clinical characteristics of T2D subjects intensifying therapy with basal insulin, overall and by study period

A total of 171,688 T2D subjects intensifying therapy with basal insulin were included in the analysis. They all received a first prescription for basal insulin and had not previously used either rapid insulin or mix-insulin. Clinical and biochemical characteristics of study subjects at the time of intensification of therapy, overall and in each 5-year period under consideration are shown in Table 1.

**Table 1:** Clinical characteristics of T2D subjects intensifying therapy with basal insulin, overall and by study period

	Overall	2005–2009	2010–2014	2015–2019
*N*	171,688	29,072	58,201	84,415
Age (years)	67.7 ± 12.2	66.9 ± 11.1	67.4 ± 12.2	68.1 ± 12.5
Men *n* (%)	96,257 (56.1)	15,342 (52.8)	32,651 (56.1)	48,264 (57.2)
Duration of diabetes (years)	9.1 ± 7.9	11.4 ± 8.4	8.8 ± 7.8	7.7 ± 7.2
BMI (Kg/m^2^)	29.7 ± 6.6	29.4 ± 5.5	29.8 ± 6.5	29.8 ± 7.1
Weight (kg)	81.0 ± 17.9	79.1 ± 16.5	80.9 ± 17.7	81.6 ± 18.4
HbA1c (%)	8.9 ± 1.8	9.0 ± 1.7	9.0 ± 1.9	8.7 ± 1.9
Subjects with HbA1c ≤ 7.0% *n* (%)	12,703 (15.1)	1865 (10.4)	4122 (14.1)	6716 (18.3)
Subjects with HbA1c > 8.0% *n* (%)	54,724 (65.3)	12,766 (71.2)	19,852 (67.8)	22,106 (60.3)
Systolic blood pressure (mmHg)	137.3 ± 19.6	140.6 ± 19.7	137.6 ± 19.7	136.1 ± 19.3
Diastolic blood pressure (mmHg)	78.3 ± 10.2	79.7 ± 9.9	78.4 ± 10.2	77.6 ± 10.3
Total cholesterol (mg/dl)	181.2 ± 43.6	189.1 ± 41.4	182.8 ± 43.7	176.1 ± 43.9
HDL-cholesterol (mg/dl)	46.9 ± 13.0	47.9 ± 13.2	46.7 ± 13.0	46.5 ± 12.8
LDL-cholesterol (mg/dl)	102.4 ± 36.3	109.5 ± 35.4	103.9 ± 36.4	97.7 ± 36.0
Triglycerides (mg/dl)	168.0 ± 118.0	167.7 ± 115.0	170.1 ± 120.4	166.4 ± 117.3
eGFR (ml/min/1.73 m^2^)	68.6 ± 28.8	71.8 ± 25.4	67.3 ± 30.8	68.5 ± 28.3
Subjects with eGFR < 30.0 ml/min/1.73 m^2^ *n* (%)	5335 (9.8)	544 (5.7)	2311 (12.2)	2480 (9.6)
Subjects with eGFR 30.0–59.9 ml/min/1.73 m^2^ *n* (%)	14,083 (26.0)	2270 (23.8)	4705 (24.9)	7108 (27.5)
Subjects with eGFR 60.0–89.9 ml/min/1.73 m^2^ *n* (%)	20,293 (37.4)	4250 (44.5)	6743 (35.7)	9300 (36.0)
Subjects with eGFR ≥ 90.0 ml/min/1.73 m^2^ *n* (%)	14,547 (26.8)	2489 (26.1)	5127 (27.1)	6931 (26.8)

Data are mean ± SD, *n*, %

*eGFR* estimated Glomerular Filtration Rate (CKD-EPI formula)

Among subjects intensifying therapy with basal insulin for the first time, from the first to the last quinquennium, we registered a progressive mild increase in the percentage of men (from 52.8 to 56.1% to 57.2%) and of mean age (from 66.9 ± 11.1 to 67.4 ± 12.2 to 68.1 ± 12.5 years). Conversely, the duration of diabetes at the time of prescribing basal insulin decreased progressively (from 11.4 ± 8.4 to 8.8 ± 7.8 and to 7.7 ± 7.2 years), suggesting a timely and more accurate prescribing attitude of Italian diabetologists.

Consistently, although mean HbA1c levels at the time of insulin prescription only modestly decreased over time, the percentage of subjects with HbA1c ≤ 7% at the time of insulin initiation increased from 10.4 to 14.1%, to 18.3%, and specularly, the percentage of subjects with HbA1c levels > 8% progressively decreased overtime (from 71 to 68 to 60%).

Overall, T2D subjects starting basal insulin presented out-of-target mean BP and lipid values. Clinical characteristics did not change in the three study periods, although major cardiovascular risk factors, such as BP, total cholesterol and LDL-Cholesterol levels, improved overtime.

On the contrary, mean values of body weight (79.1 ± 16.5 kg) and BMI (29.4 ± 5.5 kg/m^2^) which were diagnostics for an overweight condition in the first examined 5-year period, remained almost constant in the following two 5-year periods (body weight 80.9 ± 17.7 and 81.6 ± 18.4, respectively; BMI 29.8 ± 6.5 and 29.8 ± 7.1 kg/m^2^, respectively).

As for renal function, among those starting basal insulin, 36% had an impaired eGFR (< 60 ml/min/1.73 m^2^); in particular, 9.8% had eGFR < 30 ml/min/1.73 m^2^, 26% had eGFR 30–60 ml/min/1.72 m^2^, 37.4% had eGFR values 60–90 ml/min/1.73 m^2^ and 26.8% had eGFR > 90 ml/min/1.73 m^2^. Among new basal insulin users, there was a progressive increase overtime of those with moderate renal impairment (eGFR values 30–60 ml/min/1.73 m^2^) and a concomitant decrease of those with a mild eGFR decrease (eGFR 60–90 ml/min/1.73 m^2^) (Table 1).

Glucose-lowering agents prescribed in combination with basal insulin, overall and by study period, are shown in Table 2.

**Table 2:** Glucose-lowering agents prescribed in combination with basal insulin, overall and by study period

Glucose-lowering drugs	Overall	2005–2009	2010–2014	2015–2019
Metformin *n* (%)	117,251 (68.3)	19,349 (66.6)	39,447 (67.8)	58,455 (69.2)
Sulphonylureas *n* (%)	57,275 (33.4)	14,587 (50.2)	22,057 (37.9)	20,631 (24.4)
Glinides *n* (%)	25,394 (14.8)	6099 (21.0)	12,878 (22.1)	6417 (7.6)
Pioglitazone *n* (%)	5908 (3.4)	756 (2.6)	2366 (4.1)	2786 (3.3)
Acarbose *n* (%)	5423 (3.2)	770 (2.6)	2181 (3.7)	2472 (2.9)
DPP-4in *n* (%)	25,716 (15.0)	29 (0.1)	5867 (10.1)	19,820 (23.5)
GLP1-RAs *n* (%)	7309 (4.3)	37 (0.1)	1507 (2.6)	5765 (6.8)
SGLT-2in *n* (%)	8402 (4.9)	1 (0.003)	5 (0.008)	8396 (9.9)

*DPP-4in* dipeptidyl peptidase-4 inhibitors, *GLP1-RAs* Glucagon-like peptide-1 receptor agonists, *SGLT-2in* Sodium-glucose co-transporter-2 (SGLT-2 inhibitors

Metformin was the most prescribed oral agent (over 66%) in this population of T2D subjects, and its use remained constant over the three 5-year periods, while the use of glinides (from 21 to 7.6%) and sulfonylureas (from 50 to 24%) significantly decreased overtime. Acarbose and pioglitazone were overall infrequently prescribed (about 3% both) in the three 5-year period, while innovative drugs prescription rates progressively increased across the three 5-year periods. Notably, in the last quinquennium, basal insulin was prescribed in association with DPP4i in 23.5% of T2D subjects, with GLP1-RA in 6.8%, and SGLT2i in 9.9% (Table 2).

### Clinical characteristics of T2D subjects intensifying therapy with fast-acting insulin, overall and by study period

Clinical characteristics of T2D subjects on basal insulin intensifying therapy by adding fast-acting insulin, overall and by study period are shown in Table 3. All T2D subjects analyzed in this group were on basal insulin (± OHA) and intensified therapy with fast-acting insulin; Table 3 shows clinical and biochemical characteristics of study subjects at the time of intensification of therapy, in each 5-year period.

**Table 3:** Clinical characteristics of T2D subjects on basal insulin ± OHA intensifying therapy with fast acting insulin, overall and by study period

	Overall	2005–2009	2010–2014	2015–2019
*N*	137,225	34,650	54,291	48,284
Age (years)	67.2 ± 12.2	66.1 ± 11.0	67.3 ± 12.2	67.9 ± 13.1
Men *n* (%)	74,826 (54.5)	17,751 (51.2)	29,893 (55.1)	27,182 (56.3)
Duration of diabetes (years)	12.4 ± 9.1	14.5 ± 9.3	11.9 ± 9.0	10.4 ± 8.5
BMI (kg/m^2^)	29.8 ± 9.2	29.9 ± 9.1	29.8 ± 10.1	29.7 ± 8.2
Weight (kg)	80.4 ± 17.5	80.0 ± 16.5	80.4 ± 17.3	80.8 ± 18.2
HbA1c (%)	9.1 ± 2.1	8.9 ± 1.8	9.2 ± 2.2	9.1 ± 2.3
Subjects with HbA1c ≤ 7.0% *n* (%)	10,145 (15.1)	2700 (13.2)	3730 (14.8)	3715 (17.1)
Subjects with HbA1c > 8.0% *n* (%)	42,889 (63.6)	13,259 (64.8)	16,448 (65.2)	13,182 (60.8)
Systolic blood pressure (mmHg)	137.0 ± 20.4	140.0 ± 20.1	136.9 ± 20.5	134.8 ± 20.1
Diastolic blood pressure (mmHg)	77.6 ± 10.5	78.9 ± 10.0	77.3 ± 10.5	76.8 ± 10.6
Total cholesterol (mg/dl)	184.2 ± 46.3	190.4 ± 44.0	183.9 ± 46.7	179.1 ± 47.1
HDL-cholesterol (mg/dl)	47.8 ± 14.5	49.4 ± 15.0	47.4 ± 14.5	46.8 ± 13.9
LDL-cholesterol (mg/dl)	104.5 ± 37.7	109.7 ± 36.3	104.2 ± 37.8	100.1 ± 38.2
Triglycerides (mg/dl)	167.9 ± 126.5	165.5 ± 122.8	169.5 ± 128.3	167.9 ± 127.4
eGFR (ml/min/1.73 m^2^)	65.7 ± 29.2	66.6 ± 26.6	64.7 ± 30.9	66.2 ± 28.9
Subjects with eGFR < 30.0 ml/min/1.73 m^2^ *n* (%)	5226 (12.0)	1028 (9.5)	2455 (14.4)	1743 (11.2)
Subjects with eGFR 30.0–59.9 ml/min/1.73 m^2^ *n* (%)	12,625 (29.1)	3175 (29.3)	4715 (27.7)	4735 (30.4)
Subjects with eGFR 60.0–89.9 ml/min/1.73 m^2^ *n* (%)	15,336 (35.3)	4261 (39.4)	5712 (33.5)	5363 (34.4)
Subjects with eGFR ≥ 90.0 ml/min/1.73 m^2^ *n* (%)	10,238 (23.6)	2358 (21.8)	4147 (24.4)	3733 (24.0)

Data are mean ± SD, *n*, %

*OHA* oral hypoglycemic agents, *eGFR* Estimated Glomerular Filtration Rate (CKD-EPI formula)

From 2005 to 2019, a total of 137,225 T2D subjects on basal insulin intensified therapy with fast-acting insulin and were included in this analysis.

Both the percentage of men (from 51.2 to 56.3%) and mean age (from 66.1 ± 11.0 to 67.9 ± 13.1 years) progressively increased, from the first to the last quinquennium, while T2D duration at the time of starting fast-acting insulin progressively decreased overtime (from 14.5 ± 9.3 to 10.4 ± 8.5 years).

Overall, mean HbA1c levels at the time of starting fast-acting insulin were similar to that of T2D subjects starting basal insulin (9.1 ± 2.1% vs 8.9 ± 1.8%, respectively). Notably, among those first prescribed with fast-acting insulin, the percentage of subjects with at-target values (HbA1c ≤ 7%) increased from 13.2 to 17.1% and, vice-versa, the percentage of subjects with HbA1c > 8% slightly decreased (from 64.8 to 60.8%).

In the group of T2D subjects starting fast-acting insulin in add on to basal insulin, there was an overall improvement overtime of major risk factors; however, mean body weight (80.4 ± 17.5 kg) and BMI (29.9 ± 9.1 kg/m^2^) values, close to the cut-off for the diagnosis of obesity, remained almost constant in the three 5-year periods under examination (Table 3).

About renal function, among subjects starting fast-acting insulin, 41% had an impaired eGFR; in particular, 12% had eGFR < 30 ml/min/1.73 m^2^, 29.1% had eGFR 30–60 ml/min/1.73 m^2^, 35.3% had eGFR 60–90 ml/min/1.73m^2^ and 23.6% had eGFR > 90 ml/min/1.73 m^2^. Mean eGFR values were 65.7 ± 29.2 ml/min/1.73 m^2^.

The percentage of subjects with eGFR between 60 and 90 ml/min was 39.4% in the first examined quinquennium and 34.4% in the most recent one, while the distribution of subjects with the other eGFR classes did not change substantially overtime.

Glucose-lowering agents prescribed in combination with the start of multiple insulin regimens (fast-acting insulin in add-on to basal insulin), overall and by study period are shown in Table 4.

**Table 4:** Glucose-lowering agents prescribed in combination with fast acting insulin and basal insulin, overall and by study period

Glucose-lowering drugs	Overall	2005–2009	2010–2014	2015–2019
Metformin *n* (%)	33,170 (24.2)	8114 (23.4)	13,179 (24.3)	11,877 (24.6)
Sulphonylureas *n* (%)	3547 (2.6)	1342 (3.9)	1595 (2.9)	610 (1.3)
Glinides *n* (%)	2422 (1.8)	591 (1.7)	1308 (2.4)	523 (1.1)
Pioglitazone *n* (%)	657 (0.5)	156 (0.5)	293 (0.5)	208 (0.4)
Acarbose *n* (%)	1013 (0.7)	243 (0.7)	484 (0.9)	286 (0.6)
DPP-4in *n* (%)	1159 (0.8)	4 (0.0)	667 (1.2)	488 (1.0)
GLP1-RAs *n* (%)	168 (0.1)	0 (0.0)	61 (0.1)	107 (0.2)
SGLT-2in *n* (%)	2150 (1.6)	1 (0.0)	2 (0.0)	2147 (4.4)

All T2D subjects were on basal insulin (± OHA) and intensified therapy with fast acting insulin

*OHA* oral hypoglycemic agents, *DPP-4in* Dipeptidyl peptidase-4 inhibitors, *GLP1-RAs* glucagon-like peptide-1 receptor agonists, *SGLT-2in* SGLT-2 inhibitors

The use of metformin was 24.2% in this population, and it did not change over the three 5-year periods, while the use of glinides and sulfonylureas progressively decreased. Acarbose, pioglitazone, GLP-1 RAs and DPP4i were infrequently prescribed in combination with basal + fast acting insulins (< 1%), while the prescription of SGLT2i increased from 0 to 4.4% from the first to the last quinquennium.

### HbA1c levels at therapy intensification and after 12 months, by study period

**Fig. 1:**
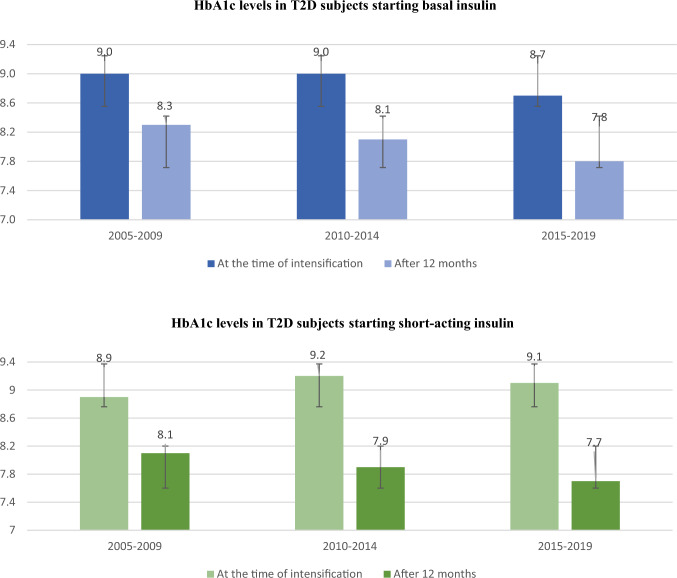
HbA1c levels (mean and standard deviation) at the time of therapy intensification and after 12 months, by study period and treatment

Mean HbA1c levels at the time of therapy intensification and after 12 months, by study period are shown in Fig. 1.

Overall, mean HbA1c levels decreased by 0.7–0.9% after 12 months from basal insulin prescription to a similar extent in the three examined periods. However, mean HbA1c levels remained above the recommended target in subjects with T2D **(**Fig. 1A).

Similarly, after 12 months from the starting of fast-acting insulin prescription, mean HbA1c levels fell by about 1 percentage point: in particular, in the 5-year period 2015–2019, mean HbA1c levels decreased from 9.1 ± 2.3% to 7.7 ± 1.4%. Even after initiating fast-acting insulin, mean HbA1c levels remained above recommended targets in the study population (Fig. 1B).

## Discussion

The appropriate positioning and timing of initiation of insulin therapy have changed over time. In this analysis from the large AMD Annals Initiative, we investigated the clinical characteristics of subjects with T2D starting either basal and/or fast-acting insulin therapy over a 15-year period span.

Our study demonstrated a modest anticipation in the prescription of basal insulin and no profound changes in that of fast-acting formulations. Despite these observations, insulin therapy is still started at high HbA1c values and, after 12 months, mean HbA1c levels are still out-of-target, thus documenting the persistence of therapeutic inertia. A consistent body of literature tried to elucidate the underlying causes of this intricate and multifaceted phenomenon, involving a complex interaction among patients’, physicians’ and health systems’ barriers [18, 25]. At this regard, a recent sophisticated analysis of the AMD Annals dataset, with the support of artificial intelligence (logic learning machine), showed that patient's dynamic glycaemic profile, instead of repeated out-of-target HbA1c values, and specifically, a meaningful difference in HbA1c values between two consecutive visits (“HbA1c gap”) is the driver for the choice of starting insulin therapy [26].

In the current analysis, the variation of clinical characteristics of subjects starting insulin therapy documented a more proactive attitude of clinicians in the first prescription of insulin therapy overtime. Thus, our data showed a progressive decrease of diabetes duration, a higher rate of subjects with HbA1c < 7% and a reduction of those with HbA1c > 8%, among T2D subjects starting insulin therapy, when comparing the last *vs.* the first quinquennium. In particular, about 40% of our patients started basal insulin when HbA1c values were still < 8%. Notably, a recent analysis on the AMD Annals dataset showed a similar trend since, in more recent years, a second or third non-insulin agents after metformin was prescribed in T2D subjects with a shorter duration of the disease and an overall better CVD profile [15]. Collectively, these findings suggest an improved approach to clinical inertia of Italian diabetologists.

On the other hand, insulin was first prescribed to subjects with far off target mean HbA1c levels (approximately of 9%) and constantly high in the explored quinquennia, with no meaningful differences between those starting basal or fast-acting formulations. This means that our patients are exposed to the risk of uncontrolled hyperglycemia for a long time. Prolonged hyperglycemia is the first and more obvious consequence of the delay in initiating basal insulin, with an increased risk for micro- and macrovascular outcomes and all-cause mortality, likely independently from other conventional risk factors [27–29]. In accordance with our findings, a study on 37.053 T2D patients from different cohorts showed that basal insulin was usually prescribed when mean HbA1c values were > 9% and after up to 24 months of uncontrolled hyperglycemia [30].

Delaying the start of insulin therapy has negative effects even in the long-term, hindering the subsequent achievement of target values, and real-world data from electronic health records have shown that baseline HbA1c levels influence the likelihood of achieving glycemic goals with various therapeutic interventions [31].

On that point, a retrospective cohort study of adults with T2D from the US and UK, confirmed that when initiation of the first injectable therapy (either basal insulin or GLP-1RA) only occurred with HbA1c considerably above target, this impeded the achievement of glucose targets [32]. Clinical inertia also resulted in worse glycemic outcomes over a 12-month period in a large medical and pharmacy claim dataset, also contributing to the increased health care utilization and costs, due to poor clinical outcomes [33].

In line with these previous observations, also in our dataset, after 12 months from the start of insulin therapy, mean HbA1c levels decreased in all the three investigated time-periods, without reaching target values for mean HbA1c, both in subjects who started basal insulin therapy and in those who started fast-acting insulin, thus confirming how the delay of intensification has important consequences on the possibility of reaching therapeutic targets subsequently. A rather discouraging result that should also be considered in the light of the general finding that overall only about half of T2D subjects reach HbA1c levels < 7%, irrespective of the treatment [8]. Specifically, for insulin-treated patients, failure to achieve therapeutic targets may also be due to insufficient insulin titration, which does not allow to adapt the therapy to individual needs. Personalization of glucose targets toward less stringent goals is another crucial issue, since elderly, frail and multimorbid patients, are those who usually starts insulin therapy. Accordingly, it should be noticed that in our study, T2D patients starting basal insulin therapy had a mean age of 68 years and a mean disease duration of 9 years, with an overall preserved renal function, whereas those starting fast-acting insulin overall showed the same mean age, with a longer diabetes duration (12 vs 9 years) and a slightly worse renal function (41% vs 36% of patients with eGFR < 60 ml/min/1.73m2).

When addressing the topic of inertia in diabetes, the importance of the management of all cardiovascular risk factors, including body weight, must also be underlined as a fundamental target of T2D care. Thus, overweight/obesity is a clinical and social problem with a growing impact in T2D patients because of its prevalence, costs and associated cardiovascular risk.

Actually, clinicians have several available therapeutic options, that must include nutritional, education and lifestyle intervention, together with the use of innovative medications. These options should be personalized, also because of the different safety profiles and degrees of effectiveness of innovative drugs [19]. Importantly, even modest weight loss can improve glucose control and cardiometabolic profile, and a 5% body weight loss through a balanced hypocaloric diet was associated with an improvement in lipid profile and markers of systemic inflammation in subjects with metabolic syndrome [34].

It is well known that the efficacy of insulin therapy is counterbalanced by the risk of hypoglycemia and undesired weight gain, and our study clearly documents that, in subjects with T2D who intensified hypoglycemic therapy with insulin therapy over three 5 years (2005–2019), the average BMI value remained high (overweight) and almost unchanged over time. This observation strongly suggests the need for a more incisive commitment of clinicians in managing body weight also in T2D starting insulin therapy.

Our study also investigated changes in the background hypoglycemic therapy in patients starting insulin. A progressive decrease in the prescription of sulphonylureas and concomitant increase of innovative drugs was registered in the third quinquennium among those starting basal insulin, although with rates still far from those recommended by diabetes guidelines. As for those starting fast-acting formulation, as expected, the rate of use of other non-insulin drugs was overall lower, and only the increase of SGLT2i (allowed in combination with basal-bolus or basal-plus insulin regimens) should be noticed. Of course, these results may reflect timely guidelines and reimbursement procedures valid at the time of insulin prescription. On the other hand, the overall stable rate of prescription of insulin therapy in T2D subjects overtime (~ 30% of T2D subjects) [8], despite the variety of currently available therapeutic options, should be related to the progressive nature of the disease, and the occurrence of beta cell dysfunction and deterioration of glucose control, especially in those with longer diabetes duration, higher baseline HbA1c and glucose levels, as well as with increased lipid, inflammatory and beta-cell dysfunction markers or those on a combination of metformin plus secretagogues [13, 14].

As for renal function, the recent availability of non-insulin drugs that can be prescribed in cases of mild–moderate renal insufficiency did not significantly change the prescribing attitude over time in patients with severe CKD in the AMD Annals. Similarly, the overall clinical characteristics of subjects starting either basal and/or fast-acting insulin, did not significantly change in the three study periods, although all major cardiovascular risk factors improved overtime. The amelioration in the control of major CVD risk factors overtime further corroborates the progressive alignment of T2D management with guidelines, although the gap remains difficult to bridge due to therapeutic inertia and the increasingly stringent recommendations [3–6], as documented by the latest analysis of the AMD Annals dataset [8].

### Limitations

Our analysis has some limitations that should be considered. First, no information on basal and fast-acting insulin titration is available, and consequently we could not explore the phenomenon of over-basalization or under-titration [35]. Moreover, a subgroup analysis of the combination of insulin with the innovative drugs, i.e., SGLT2i and GLP-1RAs, is also lacking, due to the limited number of subjects on those drugs in the considered timeframes.

## Conclusions

The choice of glucose-lowering treatment is today focused on cardiovascular and renal protection, but uncontrolled hyperglycemia often requires a targeted approach to prevent adverse outcomes, often including insulin treatment. In this large cohort of T2D subjects during a long observation period, a progressively earlier start of insulin treatment was observed, suggesting a more proactive prescriptive approach. However, the off-targets HbA1c values ​​achieved after 1 year of insulin therapy, suggest the need for greater clinical effort in T2D management.

### Supplementary Information

Below is the link to the electronic supplementary material.

Supplementary file1 (PDF 131 KB)

## Data Availability

The datasets generated during and/or analyzed in the current study are available from the corresponding author upon reasonable request.
