# Supplementary material for: Temporal trends in the starting of insulin therapy in type 2 diabetes in Italy: data from the AMD Annals initiative
Source: J Endocrinol Invest. 2024 Mar 5;47(8):2087–96. doi: 10.1007/s40618-024-02306-5 (PMC11266210; doi:10.1007/s40618-024-02306-5)
Supplement: Supplementary file 1 — Supplementary file1 (PDF 131 KB) [file 40618_2024_2306_MOESM1_ESM.pdf]

Centri partecipanti

REGIONE	AUTORI	OSPEDALE	REPARTO	CITTA'
ABRUZZO	Valeria Montani, Emanuela Cannarsa, Paola Colleluori , Vincenzo Trosini, Rosanna Pennetta , Martina Basilico	ASL di Teramo Dipartimento Discipline Mediche	UOSD Diabetologia Atri	Atri (TE)
ABRUZZO	Deamaria Piersanti, Barbara Macerola, Elisabetta Di Berardino, Alessia Di Pietro, Loredana Capaldi, Antonio Conti	ASL 1 Abruzzo - P.O. Avezzano	UOSD Diabetologia	Avezzano (AQ)
ABRUZZO	Elisabetta Straface, Mario Grasso	Distretto Sanitario di Base di Casalbordino - ASL Lanciano-Vasto-Chieti	Ambulatorio Diabetologialisa	Casalbordino (CH)
ABRUZZO	Livia Santarelli, Annamaria De Mutiis , Settimio D'Andrea, Carla Scarsellato, Antonietta Sciulli, Carla Mennella	Presidio Ospedaliero Castel di Sangro	UOSD di Diabetologia - ASL1 Abruzzo Avez zano-Sulmona-L'Aquila	Castel di Sangro (AQ)
ABRUZZO	Carla Cinzia Carrabs, Giulia Tartaro, Elena Giampietro, Ester Vitacolonna	Ospedale Clinicizzato SS Annunziata	Diabetologia del Policlinico Chieti	Chieti (CH)
ABRUZZO	Maria D'Aurizio	PTA Presidio Territoriale di Assistenza Gissi	Reparto di Diabetologia	Gissi (CH)
ABRUZZO	Sara Nazzarena Morgante, Antonella Zugaro, Marco Giorgio Baroni	Ospedale San Salvatore - L'Aquila ASL 1 Abruzzo	Diabetologia e Malattie Metaboliche	L'Aquila (AQ)
ABRUZZO	Daniela Antenucci, Fiore Pelliccione, Tiziana Rapino, Alessio Martorella , Annamaria Crognale, Morena Di Paolo, Graziella Martelli, Pamela De Giosa , Giovanna Angelicola, Ylenia Natarelli, Angela Di Federico	Ospedale F. Renzetti, ASL 2 Lanciano-Vasto-Chieti	Diabetologia e Malattie Metaboliche	Lanciano (CH)

REGIONE	AUTORI	OSPEDALE	REPARTO	CITTA'
ABRUZZO	Agostino Consoli, Gloria Formoso, Giuliana La Penna, Fabrizio Febo, Sara Coluzzi	AUSL Pescara Ospedale Civile Santo Spirito	UOC Territoriale Endocrinologia e Metabolismo	Pescara (PE)
ABRUZZO	Angelo Cosimo Gioia, Benedetta Martin	ASL Pescara Presidio Ospedaliero di Popoli	Ambulatorio Diabetologia UOC Medicina Interna	Popoli (PE)
ABRUZZO	Elisabetta Straface, Concetta Racciatti	ASL Lanciano Vasto Chieti	Ambulatorio diabetologico Distretto San Salvo	San Salvo (CH)
ABRUZZO	Rossella Romano, Patrizia Di Fulvio, Antonio Macrillante, Maria Laura Daniele	ASL 4 Teramo Ospedale Val Vibrata	UOS Diabetologia	Sant' Omero (TE)
ABRUZZO	Livia Santarelli, Annamaria De Mutiis, Luigina Ventresca, Debora Gambina, Giovanni Cavaliere, Luigina Spadorcia	Ospedale Civile SS Annunziata - ASL1 Abruzzo Avezzano-Sulmona-L'Aquila	U.O. di Diabetologia - Sulmona	Sulmona (AQ)
ABRUZZO	Mariarosaria Squadrone, Grazia Giovanna Laverghetta, Maria D'Aurizio, Concetta Massa, Donatella Luciani	Nucleo Operativo Distrettuale Vasto	Servizio Diabetologico C/0 Nod Vasto 1 piano	Vasto (CH)
ABRUZZO	Marilena Olivieri	Distretto Sanitario di Base di Villa Santa Maria - ASL Lanciano-Vasto-Chieti	Ambulatorio di Diabetologia e Endocrinologia	Villa Santa Maria (CH)
CALABRIA	Paola Sarnelli, Luigi Puccio	Azienda Ospedaliera Pugliese-Ciaccio	SOC Diabetologia - Endocrinologia	Catanzaro (CZ)
CALABRIA	Rosanna Piro	Ospedale San Francesco di Paola	Reparto di Diabetologia	Paola (CS)
CALABRIA	Eugenio Alessi, Concetta Nadia Aricò, Desiree Cannizzaro, Claudia Ferraro, Maria Angela Sculli	A.O. Bianchi Melacrino Morelli Presidio Ospedaliero Morelli	UOC Diabetologia ed Endocrinologia	Reggio Calabria (RC)
CALABRIA	Celestino Giovannini, Daniela Cristiano, Fortunata Pontari	ASP 5 Reggio Calabria Polo Sanitario Nord	Servizio di Diabetologia	Reggio Calabria (RC)
CALABRIA	Giovanni Perrone, Alessandra Paris Paris	Polo Sanitario RC-Sud ASP 5	Servizio Territoriale di Diabetologia	Reggio Calabria (RC)

REGIONE	AUTORI	OSPEDALE	REPARTO	CITTA'
CALABRIA	Anton Giulio Ametrano, Alfonso Mele, Mariagrazia Restuccia, Mariangela Rubino, Delia Francesca Carbotti, Giuseppe Armentano	Centro Diabetologico DEA - ASP Cosenza	Accreditato SSN - ASP Cosenza	Rossano Calabro (CS)
CAMPANIA	Franca Serino, Andrea Del Buono	Centro Diabetologico di Cellole ASL Caserta Distretto 14	Centro Diabetologico di Cellole	Cellole (CE)
CAMPANIA	Maria Carmen Passariello, Mario Laudato	ASL Caserta Distretto Sanitario 13 - Maddaloni	Ambulatorio di diabetologia	Maddaloni/San Felice A Cancello (CE)
CAMPANIA	Claudio Lambiase, Teresa Di Vece, Luigi Sardelli, Gabriella Nosso, Luigi Gargiulo, Maurizio Mauriello, Massimo Fezza, Carmela Giordano, Flora Leo, Linda D'Amato	Ospedale Curteri G. Amico DS 67 - ASL SA	Centro Diabetologico DS 67 Mercato S. Severino	Mercato S. Severino (SA)
CAMPANIA	Stefano De Riu	ASL NA 1 - Distretto sanitario 33		Napoli (NA)
CAMPANIA	Adriano Gatti	Presidio Ospedaliero San Gennaro - ASL NA1 Centro	Centro di Diabetologia 4 Piano	Napoli (NA)
CAMPANIA	Claudio Lambiase, Stefano Masi, Andrea Renzullo, Lea Lo Conte, Giovanni Carella, Anna Nasti, Carmela Peluso, Lucia D'Elia, Ylenia Nicole Pellegrino, Vincenzo De Caprio	Asl di Salerno	C.D.A. Distretto Sanitario Nocera Inferiore	Nocera Inferiore (SA)
CAMPANIA	Emilia Martedi'	Centro Diagnostico San Ciro	Centro AID Portici	Portici (NA)
CAMPANIA	Gelsomina Capuano, Alessandra Cantillo, Lea Lo Conte , Giovanni Attanasio	ASL Salerno Centro Diabetologico Distretto 66 Ospedale Vernieri	Centro Diabetologico	Salerno (SA)
CAMPANIA	Francesca Innelli, Angela Cassese, Piera Grammaldo, Angelo Vistocco, Valentino De Stefano, Luigi Scevola,	Ambulatorio Diabetologia Distretto Sanitario 70	Ambulatorio Diabetologia	Vallo della Lucania (SA)

REGIONE	AUTORI	OSPEDALE	REPARTO	CITTA'
	Angela Cetrangolo, Emanuela Petraglia			
EMILIA ROMAGNA	Gilberto Laffi, Pagotto Uberto , Michele Salvatore Grimaldi, Valentina Lo Preiato, Simona Moscatiello, Danilo Ribichini	AOU di Bologna Policlinico S. Orsola Malpighi	Unita' Operativa di Diabetologia	Bologna (BO)
EMILIA ROMAGNA	Ugo Aldo Pagliani , Monica Camporesi, Giorgia Prampolini, Marzia Turilli, Licia Notari	Ospedale di Castelnovo ne Monti	Diabetologia	Castelnovo ne' Monti (RE)
EMILIA ROMAGNA	Vincenzo Monda	Ospedale di Cento - Casa della Comunità di Bondeno	UOC Diabetologia Territoriale AUSL Ferrara	Cento - Bondeno (FE)
EMILIA ROMAGNA	Diletta Ugolotti, Elisa Usberti	AUSL Parma Ambulatorio Casa della Salute Collecchio	Ambulatori Diabetologici Distretto Sud - Est	Collecchio (PR)
EMILIA ROMAGNA	Carlo Percudani	AUSL PARMA Polo Sanitario di Colorno	Ambulatorio Diabetologico	Colorno (PR)
EMILIA ROMAGNA	Elisabetta Bergami	Ospedale di Comacchio - Casa della Comunità di Codigoro	UOC Diabetologia Territoriale AUSL Ferrara	Comacchio - Codigoro (FE)
EMILIA ROMAGNA	Anna Rita Carli	Casa della Comunità di Copparo	UOC Diabetologia Territoriale AUSL Ferrara	Copparo (FE)
EMILIA ROMAGNA	Bruna Milli, Silvia Pilla, Petros Tsamatropoulos, Giulia Bellei, Daniela Di Renzo, Marinella Angeli, Annalisa Iotti, Elena Carboni, Paola Caretta, Valeria Vezzani, Morena Marmiroli, Susanna Rovesti, Elisa Monzali	Ospedale di Correggio	Diabetologia	Correggio (RE)
EMILIA ROMAGNA	Maria Simona Termine	PO di Faenza - AUSL della Romagna	UO Diabetologia Ravenna/Faenza, Dip. Internistico	Faenza (RA)
EMILIA ROMAGNA	Marcello Monesi, Francesca Lugli, Bellio Mariaenrica, Serena Galvan, Elisa Dinatolo	Casa della Comunità "Cittadella San Rocco" Ferrara	UOC Diabetologia Territoriale AUSL Ferrara	Ferrara (FE)

REGIONE	AUTORI	OSPEDALE	REPARTO	CITTA'
EMILIA ROMAGNA	Elisa Gatti, Nicoletta Orlandi, Antonella Guberti	Ospedale di Fidenza	U.O.Medicina Interna	Fidenza (PR)
EMILIA ROMAGNA	Bruna Milli, Silvia Pilla, Giulia Bellei, Petros Tsamatropoulos, Paola Caretta, Daniela Di Renzo, Valeria Vezzani, Morena Marmiroli, Susanna Rovesti, Elisa Monzali, Marinella Angeli, Annalisa Iotti, Elena Carboni	Ospedale di Guastalla	Diabetologia	Guastalla (RE)
EMILIA ROMAGNA	Rita Manini, Anna Vacirca	Ospedale Civile Nuovo di Imola	Dipartimento Medico Oncologico	Imola (BO)
EMILIA ROMAGNA	Diletta Ugolotti, Elisa Usberti, Sonia Erta, Tiziana Cadossi, Daniela Pedrini	Ausl Parma Ambulatorio Casa della Salute di Langhirano	Ambulatorio Diabetologia	Langhirano (PR)
EMILIA ROMAGNA	Alessandra Luberto	PO di Lugo - AUSL della Romagna	UO Diabetologia Ravenna/Lugo, Dip. Internistico	Lugo (RA)
EMILIA ROMAGNA	Massimo Michelini, Rosa Maria Trianni, Elena Capuano, Francesca Borghi, Fiorenza Delloste, Serena Davoli, Marika Iemmi, Paola Pedrini, Monica Ronchetti, Annalisa Tagliavini, Paola Ferrari	Ospedale di Montecchio	Diabetologia	Montecchio (RE)
EMILIA ROMAGNA	Alessandra Dei Cas, Riccardo Bonadonna, Raffaella Aldigeri, Monica Antonini, Angela Vazzana, Valentina Moretti	AOUPR Azienda Ospedaliera Universitaria Parma - Dip. di Medicina generale e specialistica	UOC di Endocrinologia e Malattie del metabolismo	Parma (PR)
EMILIA ROMAGNA	Diletta Ugolotti, Clelia Di Secli, Antonella Guberti	AUSL Parma Polo Sanitario Pintor Molinetto Parma	Ambulatorio Diabetologico	Parma (PR)
EMILIA ROMAGNA	Silvia Haddoub, Michele Riva, Maria Grazia Magotti, Maria Maddalena Micheli	Azienda Ospedaliero-Universitaria di Parma	Reparto di Trattamento intensivo Del Diabete e delle sue Complicanze	Parma (PR)
EMILIA ROMAGNA	Biagio Oliviero	Ospedale di Argenta -	UOC Diabetologia	Portomaggiore -

REGIONE	AUTORI	OSPEDALE	REPARTO	CITTA'
		Casa della Comunità di Portomaggiore	Territoriale AUSL Ferrara	Argenta (FE)
EMILIA ROMAGNA	Paolo Di Bartolo, Francesca Pellicano, Cipriana Sardu, Chiara Caselli, Mazzotti Arianna	AUSL della Romagna	REPARTO DI DIABETOLOGIA	Ravenna (RA)
EMILIA ROMAGNA	Romina Amodeo, Lisa Bonilauri , Maria Cardaci, Eliana Gardini, Alessandro Lo Ioco, Roberta Lunghi, Laura Lombardi, Susanna Gelosini , Elisa Manicardi, Maria Gloria Mercati, Rita Montedoro, Roberta Prandi, Sara Pingani, Prisco Sbordone, Rosa Maria Trianni, Miriam Parisi, Francesca Palmieri, Marta Ghinelli, Eles Notari, Elisa Berri	Casa della Salute, Distretto di Reggio Emilia	Diabetologia	Reggio Emilia (RE)
EMILIA ROMAGNA	Ugo Aldo Pagliani, Monica Camporesi, Alfredo Zappavigna, Antonella Rabitti, Anna Maria Ferraioli, Susanna Valenti, Simona Bodecchi	AUSL di Reggio Emilia-Ospedale di Scandiano	Diabetologia	Scandiano (RE)
EMILIA ROMAGNA	Elisa Usberti, Diletta Ugolotti, Daniela Pedrini	AUSL Parma Ambulatorio casa della Salute di Traversetolo	Ambulatorio di Diabetologia	Traversetolo (PR)
FRIULI VENEZIA GIULIA	Mario Velussi	Casa di Cura Pineta del Carso	Ambulatorio di Diabetologia	Aurisina (TS)
FRIULI VENEZIA GIULIA	Giuseppe Felace, Manola Nicoletti, Daria Albini, Fabrizio Santoro, Elisa Norio, Lorj Mongiat, Maria Elena Salvador, Nicoletta Petracco	ASFO (Azienda Sanitaria Friuli Occidentale) Ospedale S. Maria dei Battuti di San Vito al Tagliamento - sede di Maniago	Ambulatorio Diabetologico	Maniago (PN)
FRIULI VENEZIA GIULIA	Paolo Bordin , Luciana Dotto, Maurizio Sancandi , Elena Rossa	AAS n.3 Alto Friuli - Collinare-Medio Friuli O.C. Sant' Antonio	Ambulatorio di Diabetologia - UO Medicina	S. Daniele del Friuli (UD)
FRIULI VENEZIA GIULIA	Patrizia Li Volsi, Paola Pusiol	Ospedale Civile Di Sacile AAS5	Reparto di Diabetologia	Sacile (PN)

REGIONE	AUTORI	OSPEDALE	REPARTO	CITTA'
		Pordenone		
FRIULI VENEZIA GIULIA	Manola Nicoletti, Martina Grando, Giuseppe Felace, Rita Centis	Azienda Ospedaliera Universitaria S. Maria della Misericordia sede di San Vito al Tagliamento	Ambulatorio di Diabetologia	San Vito al Tagliamento (PN)
FRIULI VENEZIA GIULIA	Giuseppe Felace, Daria Albini, Fabrizio Santoro, Massimo Rondana, Agnese Fasano, Ilenia Donaduzzi, Tiziana Del Savio	ASFO (Azienda Sanitaria Friuli Occidentale) - Presidio Ospedaliero S.Giovanni dei Battuti	Ambulatorio di Diabetologia	Spilimbergo (PN)
FRIULI VENEZIA GIULIA	Riccardo Candido, Anna Vittoria Ciardullo, Chiara Gottardi, Elena Manca, Alessandra Petrucco, Michela Casson, Elisabetta Tommasi, Katja Tercelj, Augusta Toso, Silvana Cum, Elisa Del Forno, Elena Mellini, Veronica Fragiacomo, Fabrizia Banello, Anna Maria Valiani, Alessandra Bresciani, Cinzia Sain	Azienda Sanitaria Universitaria Giuliano Isontina	S.S. Centro Diabetologico	Trieste (TS)
FRIULI VENEZIA GIULIA	Silvia Galasso, Sandra Agus, Maria Carpentieri, Silvia Maria Sciannimanico, Antonio Stefano Salcuni, Cristina Sartori, Francesca Vidotti, Graziana Fabbro, Rita Brovedani, Angela Retugliano, Anna Rita Paccini, Giovanna Cassan, Lucia Caputo, Marina Armellini	Azienda Sanitaria Universitaria Friuli Centrale (ASUFC)	SOC di Endocrinologia - ASUFC	Udine (UD)
LAZIO	Claudio Grande	ASL Viterbo - Ospedale di Acquapendente	Ambulatorio di diabetologia	Acquapendente (VT)
LAZIO	Grazia Pia Ricciardi	ASL Latina Distretto 1 Aprilia	Servizio diabetologia	Aprilia (LT)
LAZIO	Maria Cristina Ribaudo, Laura Proietti	ASL RM 4	Diabetologia	Capena (RM)

REGIONE	AUTORI	OSPEDALE	REPARTO	CITTA'
	Pannunzi, Daniele Follieri , Maria Paola Laria , Elisabetta Meloni, Stefania Ciriello, Anna Lorenzetti			
LAZIO	Graziano Santantonio, Alessandra Zappaterreno, Lucrezia Russo, Olimpia Bitterman, Lina Lottatori, Elisa Costanzo, Debora Fabbretti, Valentina Arezzini, Valeria De Persio, Valentina Di Fiordo, Daniele Follieri	Presidio Ospedaliero San Paolo - Civitavecchia	U.O.S.D. Diabetologia	Civitavecchia (RM)
LAZIO	Roberta Gaudioso, Vittoria Vaccari	ASL Roma 5 - Ospedale di Colleferro	UOC Medicina Interna e Geriatria Ambulatorio di Diabetologia	Colleferro (RM)
LAZIO	Francesco De Meo, Elisa Forte, Daniela Fiore, Generosa Pannozzo	Ospedale di Fondi	Reparto di Diabetologia	Fondi (LT)
LAZIO	Francesco De Meo, Elisa Forte, Rossella Fabiano, Daniela Fiore, Claudia Iannone	Presidio Ospedaliero di Gaeta	SC Diabetologia ed Endocrinologia	Gaeta (LT)
LAZIO	Raffaele Scalpone , Patrizio Tatti, Gregorio Versace	INI Istituto Neurotraumatologico Italiano	Ambulatorio di Diabetologia/Reparto di Medicina Generale	Grottaferrata (RM)
LAZIO	Lorena Mancini, Giulia Bassotti, Danuta Wolosinska, Gloria Cornacchiola, Di Biagio Debora, Donnino Rita, Vallesi Celeste, Follieri Daniele, Di Fiordo Valentina	ASL Roma 4 - Casa della Salute Ladispoli/Cerveteri	Diabetologia	Ladispoli (RM)
LAZIO	Angela Carlone, Alessandra Di Flaviani, Claudio Caccamo , Alessia Ventricini , Lucia Fontana	ACISMOM LATINA	Centro Diabetologico Latina	Latina (RM)

REGIONE	AUTORI	OSPEDALE	REPARTO	CITTA'
LAZIO	Frida Leonetti	Ospedale Santa Maria Goretti	UOC di Diabetologia Universitaria	Latina (RM)
LAZIO	Anna Rita Aleandri, Silvia Caprioli, Giuseppina Beretta Anguissola, Maria Virginia Guidi, Fabrizia Toscanella, Maria Grazia Pipitone, Maria Rosaria Faraglia, Verena Lilli, Cristina Giagnoli, Michela Di Venanzio, Maria Cristina Raggi, Eleonora Pomponi	Ospedale San Camillio De Lellis -ASL Rieti	Medicina 2 - Diabetologia	Rieti (RI)
LAZIO	Maria Letizia Bruschi, Loredana Pomponi	Ambulatorio Diabetologia ASL Roma 2	Ambulatorio Diabetologia	Roma (RM)
LAZIO	Ernesto Maddaloni, Chiara Moretti, Luca D'Onofrio, Carmen Mignogna, Antonio Siena, Raffaella Buzzetti	Azienda Ospedaliera Universitaria Policlinico Umberto I	UOD di Diabetologia	Roma (RM)
LAZIO	Paola D'Angelo, Angela Del Prete, Teresa Mondello, Severino Malizia, Salvatore Valentino, Maria Altomare, Santina Abbruzzese, Silvia Carletti, Roberta Lancione, Teresa Di Fiore, Veronica Tamburri, Marianna Battimelli, Daniela Finora, Gabriella Ceccarelli, Roberto Gagliardi	Ospedale Sandro Pertini	UO-Diabetologia	Roma (RM)
LAZIO	Alessandra Di Flaviani, Stefania Angotti, Barbara Carabba, Maddalena Ragazzo, Sara Sterpetti, Francesco Saverio Floridi	ACISMOM Palmiro Togliatti	Centro Diabetologico Palmiro Togliatti	Roma (RM)
LAZIO	Valentina Izzo, Alessandra Di Flaviani, Rossella	ACISMOM CONCORDIA	Centro Diabetologico Concordia	Roma (RM)

REGIONE	AUTORI	OSPEDALE	REPARTO	CITTA'
	Fabiano , Stefano Colangelo , Roberto Gagliardi , Caterina Saponara , Leonardo Lancia , Fabio Colletti			
LAZIO	Roberta Pisano, Maria Cristina Gentile, Diana Corradini, Sofia De Taddeo, Francesca Piccirilli, Maria Neve	ACISMOM NEGRO	Centro Diabetologico Camillo Negro	Roma (RM)
LAZIO	Danila Fava, Maria Cassone Faldetta, Fulvia De Luca, Assunta Santonati, Giuliana Leacche, Anna Conidi, Cristina Sacchini, Rossella Guarino, Vanessa Maini, Valentina Perfili, Anna Tesei	A.O. S. Giovanni Addolorata Presidio Ospedaliero Santa Maria	UOSD Endocrinologia e Diabetologia	Roma (RM)
LAZIO	Simona Frontoni, Fabiana Picconi, Patrizia Borboni, Federica De Marco, Marika Menduni, Alessio Maiorino, Sofia De Taddeo, Benedetta Russo, Daniela Ceccaroni, Giuseppe Vancieri, Valentina Tommasi	Ospedale Fatebenefratelli Isola Tiberina - Gemelli Isola	UOC Endocrinologia, Diabetologia e Malattie Metaboliche	Roma (RM)
LAZIO	Andrea Giaccari, Teresa Mezza, Francesca Cinti, Gianfranco Di Giuseppe, Umberto Capece, Gea Ciccarelli, Michela Brunetti, Laura Soldovieri, Valentina Fragale, Ilaria Improta, Flavia Impronta, Chiara Maria Assunta Cefalo, Simona Moffa	Fondazione Policlinico Agostino Gemelli IRCCS	Centro per le malattie endocrine e metaboliche	Roma (RM)
LAZIO	Susanna Morano, Tiziana Filardi, Vittorio Venditti, Enrico Bleve, Maria Cristina Gentile, Antonella Valente	Policlinico Umberto I, Sapienza Universita' di Roma	Dipartimento di Medicina Sperimentale UOS Complicanze del diabete	Roma (RM)

REGIONE	AUTORI	OSPEDALE	REPARTO	CITTA'
LAZIO	Lelio Morviducci, Daniela Cappelloni, Anna Ciarmatori, Lina Lardieri, Ilaria Giordani, Tiziana Santucci, Gabriella Del Monte, Franca Rauseo, Patrizia Alini, Daniela Chiodi, Catia Pietrangeli, Maria Elena Antonazzi , Romina Di Mauro, Claudia Brufani	Azienda Ospedaliera San Filippo Neri	U.O.D. Diabetologia	Roma (RM)
LAZIO	Lelio Morviducci, Renato Giordano, Maria Giuseppina Migneco, Mauro Rossini, Elettra Santarelli, Natalia Visalli, Giovanna Liberati, Ida La Cesa, Patrizia Cortesi, Orietta Pannozzo, Umberto Gazzarini, Claudia Leonoro, Romina Ralli , Samanta Roma, Claudia Righini, Bruna Facchini	Ospedale Santo Spirito	U.O.C. Diabetologia	Roma (RM)
LAZIO	Lelio Morviducci, Fabiana Lanti, Sabrina Spera, Enrica Salomone, Cinzia Riccobono, Marco Pietrantoni, Roberta La Rosa, Anna D'Ubaldi	Ospedale Nuovo Regina Margherita	U.O.C. Diabetologia	Roma (RM)
LAZIO	Francesco Sabetta, Concetta Suraci, Sabrina Braucci, Laura Borgognoni, Antonella Caroli, Fabiana Trulli, Patrizia Galante , Gloriana Bergami, Letizia Mariani	Eurosanita' S.p.A. - Policlinico Casilino	Ambulatorio di Diabetologia - UO Medicina Interna	Roma (RM)
LAZIO	Maria Rosaria Nardone, Claudio Tubili, Angelo Lauria Pantano	AO San Camillo Forlanini	UOSD Diabetologia	Roma (RM)
LAZIO	Luigi Uccioli, Pasquale Di Perna, Chiara	Ospedale CTO	UOC Endocrinologia e Malattie Metaboliche	Roma (RM)

REGIONE	AUTORI	OSPEDALE	REPARTO	CITTA'
	Pecchioli, Patrizia Sperti			
LAZIO	Claudio Ventura, Vittoria Bonato, Marzia Bongiovanni, Esmeralda Borrello, Emma Condorelli, Angela Napoli, Sabrina Coen	Ospedale Israelitico	U.O.S. Endocrinologia e M. Metaboliche	Roma (RM)
LAZIO	Francesco Malci, Anita De Ciocchis, Paolo Martini, Manuela Fiducia	ASL RM G Presidio Ospedaliero A. Angelucci - Subiaco	Medicina Interna	Subiaco (RM)
LAZIO	Paolo Fiorentini	ASL Viterbo - Ospedale di Tarquinia	Ambulatorio di diabetologia	Tarquinia (VT)
LAZIO	Francesco De Meo, Elisa Forte , Daniela Fiore , Anna Pacilio	Ospedale di Terracina Latina	Reparto di Diabetologia	Terracina (LT)
LAZIO	Vincenzo Fiore, Alessandra Barucca, Giovanni Carbotta, Alberico Giustini, Antonella Poggi, Maurizio Palmieri, Cinzia Sforza, Dionisia Carinella, Antonio Angelucci, Anna De Paolis, Antonella Di Pasquali, Sonia Barraco	ASL Roma 5	UOSD Diabetologia - Endocrinologia	Tivoli (RM)
LAZIO	Michela Dainelli, Alessandra Di Flaviani, Lucia Fontana	ACISMOM Centro Diabetologico Di Viterbo	Centro Diabetologico di Viterbo	Viterbo (VT)
LAZIO	Paolo Fiorentini, Claudia Arnaldi, Venanzi Susanna, Anna Montebove, Simona Vincenti, Cinzia Fiorillo, Barbara Longo, Alfonsina Chiefari, Claudio Grande, Tittania Musella, Davide Tosini	ASL Viterbo	Centro Diabetologico Aziendale	Viterbo (VT)
LIGURIA	Eleonora Ambrosetti, Micaela Battistini, Francesca Cecoli,	ASL 3 Genovese	SSD Diabetologia Endocrinologia Malattie Metaboliche	Arenzano (GE)

REGIONE	AUTORI	OSPEDALE	REPARTO	CITTA'
	Arianna Cesarone, Paola Angela Cichero, Enrico Torre, Francesca Fabbri, Francesca Annunziata, Giovanni Careddu, Laura Veronica Camerieri, Miryam Talco, Eleonora Monti, Maurizio Patrone, Roberta Guido, Valeria Ghigliotti, Alberto Rebora			
LIGURIA	Paola Ponzani, Maria Rosaria Falivene, Danilo Conti	ASL 4 Liguria	SSD Diabetologia e Malattie Metaboliche	Chiavari (GE)
LIGURIA	Eleonora Ambrosetti, Micaela Battistini, Francesca Cecoli, Arianna Cesarone, Paola Angela Cichero, Enrico Torre, Francesca Fabbri, Francesca Annunziata, Giovanni Careddu, Laura Veronica Camerieri, Miryam Talco, Eleonora Monti, Maurizio Patrone, Roberta Guido, Valeria Ghigliotti, Alberto Rebora	ASL 3 Genovese Ambulatorio Voltri - Pegli	SSD Diabetologia Endocrinologia Malattie Metaboliche	Genova (GE)
LIGURIA	Eleonora Ambrosetti, Micaela Battistini, Francesca Cecoli, Arianna Cesarone, Paola Angela Cichero, Enrico Torre, Francesca Fabbri, Francesca Annunziata, Giovanni Careddu, Laura Veronica Camerieri, Miryam Talco, Eleonora Monti, Maurizio Patrone, Roberta Guido, Valeria Ghigliotti, Alberto Rebora	ASL 3 Genovese Palazzo della salute di Fiumara	SSD Diabetologia-Endocrinologia-Malattie Metaboliche	Genova (GE)
LIGURIA	Eleonora Ambrosetti, Micaela Battistini,	ASL 3 Genovese Ex Ospedale Nervi	SSD Diabetologia-Endocrinologia-Malattie	Genova (GE)

REGIONE	AUTORI	OSPEDALE	REPARTO	CITTA'
	Francesca Cecoli, Arianna Cesarone, Paola Angela Cichero, Enrico Torre, Francesca Fabbri, Francesca Annunziata, Giovanni Careddu, Laura Veronica Camerieri, Miryam Talco, Eleonora Monti, Maurizio Patrone, Roberta Guido, Valeria Ghigliotti, Alberto Rebora		Metaboliche	
LIGURIA	Eleonora Ambrosetti, Micaela Battistini, Francesca Cecoli, Arianna Cesarone, Paola Angela Cichero, Enrico Torre, Francesca Fabbri, Francesca Annunziata, Giovanni Careddu, Laura Veronica Camerieri, Miryam Talco, Eleonora Monti, Maurizio Patrone, Roberta Guido, Valeria Ghigliotti, Alberto Rebora	ASL 3 Genovese Poliambulatorio Largo 12 Ottobre	SSD Daibetologia Endocrinologia Malattie Metaboliche	Genova (GE)
LIGURIA	Eleonora Ambrosetti, Micaela Battistini, Francesca Cecoli, Arianna Cesarone, Paola Angela Cichero, Enrico Torre, Francesca Fabbri, Francesca Annunziata, Giovanni Careddu, Laura Veronica Camerieri, Miryam Talco, Eleonora Monti, Maurizio Patrone, Roberta Guido, Valeria Ghigliotti, Alberto Rebora	ASL 3 Genovese Ex Ospedale Sant'Antonio	SSD diabetologia Endocrinologia e Malattie Metaboliche	Recco (GE)
LIGURIA	Luca Lione, Mario Monachesi, Amelia Casalini, Simona Oddera	ASL 2 Savonese	Ambulatorio di Diabetologia Territoriale	Savona (SV)

REGIONE	AUTORI	OSPEDALE	REPARTO	CITTA'
LIGURIA	Luca Lione, Gigi Bocchio, Edmondo Bosco, Giancarlo Markic, Mario Monachesi	UCP Savona Ponente	Ambulatorio di Diabetologia	Savona (SV)
LOMBARDIA	Stefano Fazion, Marialuisa Spina, Anna Melegari, Maria Elena Cerutti	ASST-Mantova, Ambulatorio di Asola	SSD di Diabetologia e Malattie Metaboliche	Asola (MN)
LOMBARDIA	Stefano Fazion, Alessandra Malaspina, Monica Rizzardi, Loredana Beschi, Virna Pasi, Anna Agosta	ASST-Mantova, Ambulatorio di Bozzolo	SSD di Diabetologia e Malattie Metaboliche	Bozzolo (MN)
LOMBARDIA	Elena Cimino, Barbara Agosti, Sara Madaschi, Cristina Mascadri, Giulia Massari, Bernadetta Pasquino, Eugenia Resmini, Emanuela Zarra, Angela Girelli	ASST -Spedali Civili di Brescia	UOC Medicina Generale ad indirizzo Diabetologico	Brescia (BS)
LOMBARDIA	Ida Mangone, Veronica Vilei, Silvia Galliani	ASST della Brianza	SSD di Malattie Endocrine, del Ricambio e della Nutrizione	Carate (MB)
LOMBARDIA	Valeria Guazzoni, Gloria Groppelli, Andrea Carbone , Martina Molteni , Linda Minelli , Sara Lodigiani	Presidio Ospedaliero di Casalpusterlengo, ASST di Lodi	Ambulatorio di diabetologia , Presidio Ospedaliero di Casalpusterlengo, afferente all'UOS Diabetologia ed endocrinologia ASST di Lodi	Casalpusterlengo (LO)
LOMBARDIA	Stefano Fazion, Maria Luisa Spina, Anna Melegari, Maria Elena Cerutti	ASST-Mantova, Ambulatorio di Castel Goffredo	SSD di Diabetologia e Malattie Metaboliche	Castel Goffredo (MN)
LOMBARDIA	Davide Attilio Ghelfi, Giuseppina De Felice, Giovanna Squiccimarro, Andrea Magistro , Monica Castellan, Maria Iagulli, Rosaria Suriano, Laura Pessina, Sara Fabbri, Chiara	Ospedale E. Bassini ASST Nord Milano	SS di Diabetologia e Malattie Metaboliche	Cinisello Balsamo (MI)

REGIONE	AUTORI	OSPEDALE	REPARTO	CITTA'
	Lessa, Sabrina Chirone, Alberto Rocca			
LOMBARDIA	Valeria Guazzoni, Gloria Groppelli , Andrea Carbone , Martina Molteni , Linda Minelli , Sara Lodigiani	ASST di Lodi , Presidio Ospedaliero di Codogno	Ambulatori di Diabetologia , Presidio ospedaliero di Codogno, UOS di Diabetologia ed endocrinologia dell'ASST di Lodi	Codogno (LO)
LOMBARDIA	Cinzia Massafra, Maddalena Torresan, Grazia Ferro	ASST Nord Milano	Presidio dei Poliambulatori - Ambulatorio di Cologno Monzese	Cologno Monzese (MI)
LOMBARDIA	Giuseppe Carrano, Chiara Mauri, Anna Bogani, Francesca Pesenti, Annalisa Creanza, Mariagrazia Tettamanzi, Rachele Motta, Beatrice Cogo, Pozzoli Raffaella, Elli Paolo	ASST Lariana	Azienda Ospedaliera Sant'Anna - Diabetologia	Como (CO)
LOMBARDIA	Patrizia Ruggeri, Sergio Di Lembo, Rosa Moretta, Romano Persico, Cristiana Rossi, Amalia Degli Innocenti, Alida Campagnoni	ASST Presidio Ospedaliero di Cremona	Centro Diabetologico	Cremona (CR)
LOMBARDIA	Paolo Marenco, Rosana Gaiofatto, Maria Albanese, Augusto Lovagnini Scher	ASST Nord Milano	Presidio dei Poliambulatori - Ambulatorio di Cusano Milanino	Cusano Milanino (MI)
LOMBARDIA	Ida Mangone, Annalisa Giancaterini, Emanuele Spreafico, Gabriella Rizzi, Elena Lucia Colombo, Cinzia Galimberti	ASST della Brianza	SSD di Malattie Endocrine, del Ricambio e della Nutrizione	Desio (MB)
LOMBARDIA	Giuseppina Zaltieri, Donata Richini , Roberto Strazzeri	ASST Valcamonica	UOSS Diabetologia e Malattie del Metabolisimo	Esine (BS)
LOMBARDIA	Ida Mangone, Veronica Vilei, Silvia Galliani	ASST della Brianza	SSD di Malattie Endocrine, del Ricambio e della Nutrizione	Giussano (MB)

REGIONE	AUTORI	OSPEDALE	REPARTO	CITTA'
LOMBARDIA	Valeria Guazzoni, Gloria Groppelli, Andrea Carbone , Martina Molteni, Linda Minelli, Sara Lodigiani, Cristina Assandri, Simona Devecchi , Emilia Oleari, Silvia Breviglieri , Flavia Sormani, Rosalba Muleddu	ASST di Lodi, Ospedale Maggiore di Lodi	UOS Diabetologia e Endocrinologia, ASST di Lodi	Lodi (LO)
LOMBARDIA	Stefano Fazion, Francesca Saggiani, Malaspina Alessandra, Roberta Nuvolari, Maura Bosi, Alessia Sanguanini, Simonetta Chiarucci, Raffaella Antoniazzi, Paola Cristanini, Elena Pierobon, Laura Paltrinieri, Samanta Aldrovandi, Roberta Casella, Simonetta Moscatelli, Rita Nardi, Cristina Pavan	ASST-Mantova, Ospedale Carlo Poma	SSD di Diabetologia e Malattie Metaboliche	Mantova (MN)
LOMBARDIA	Luigi Sciangula, Alessandra Ciucci, Baldassare Grassa, Giacomo Sturniolo, Emanuela Simona Olivo, Maria Grazia Magni, Adele Tono, Silvana Pastori, Barbara Rovelli, Eleonora Gasparotto	Presidio Polispecialistico di Mariano Comense - ASST Lariana	Struttura Semplice InterDipartimentaleDiabetologia e Endocrinologia	Mariano Comense (CO)
LOMBARDIA	Paolo Fiorina, Maria Elena Lunati, Luciana Maria Vallone , Laura Plebani, Camilla Tinari, Edoardo Baggio	Azienda Socio Sanitaria Fatebenefratelli - Sacco/Ospedale Fatebenefratelli e Oftalmico	SSD Malattie Metaboliche - Diabetologia	Milano (MI)
LOMBARDIA	Paolo Fiorina, Paola Silvia Anna Morpurgo, Alessandra Gandolfi, Andrea Mario Bolla	Presidio Ospedaliero Macedonio Melloni - ASST Fatebenefratelli Sacco	Ambulatorio di Endocrinologia e Diabetologia	MILANO (MI)
LOMBARDIA	Paolo Fiorina, Ida Pastore, Antonio Rossi , Laura Montefusco,	ASST Fatebenefratelli Sacco - Centro Ricerca Invernizzi, Padiglione	Malattie Endocrine e Diabetologia	Milano (MI)

REGIONE	AUTORI	OSPEDALE	REPARTO	CITTA'
	Enrica Chebat, Milena Muratori	62		
LOMBARDIA	Rosa Terranova	ASST Nord Milano	Presidio dei Poliambulatori - Ambulatorio di via Livigno	MIlano (MI)
LOMBARDIA	Gabriella Piscitelli, Ivana Defronzo	ASST Nord Milano	Presidio dei Poliambulatori - Ambulatorio di Piazzale Accursio	MIlano (MI)
LOMBARDIA	Raffaella Mattioni, Simona Citterio, Rosita Stanga, Nadia Devincenzo	ASST Nord Milano	Presidio dei Poliambulatori - Ambulatorio di Via Farini	MIlano (MI)
LOMBARDIA	Mariluce Barrasso	ASST Nord Milano	Presidio dei Poliambulatori - POLIAMBULATORI O VIA MASANIELLO	Milano (MI)
LOMBARDIA	Marina Scavini, Giuseppe Ancona, Loredana Bonisolli, Amelia Caretto, Alberto Davalli, Gabriella Galimberti, Andrea Laurenzi, Sabina Martinenghi, Chiara Molinari, Matteo Rocco Pastore, Erika Pedone, Giovanna Petrella, Emanuela Setola, Emanuele Bosi, Anna Zanardini, Walter Pepe, Sonia Letizia, Anna Vigna, Chiara Sartori	Istituto Scientifico-Universitario H. San Raffaele	Diabetologia - UO Medicina Generale ad indirizzo Diabetologico o Endocrino Metabolico	Milano (MI)
LOMBARDIA	Stefano Benedini, Regina Dagani, Marco Laneri, Saibene Alessandro, Adolfo Carlo Bianchi	Clinica San Carlo - Casa di cura privata Poli specialistica S.p.a.	Sevizio di Diabetologia	Paderno Dugnano (MI)
LOMBARDIA		ASST Bergamo Est - Ospedale M.O. Locatelli	Servizio di Diabetologia	Piario (BG)
LOMBARDIA	Marica Sormani, Antonio Tempesta, Fabrizia Didoni,	Presidio Ospedaliero di Rho - ASST Rhodense	Ambulatorio di diabetologia	Rho (MI)

REGIONE	AUTORI	OSPEDALE	REPARTO	CITTA'
	Michela Gianni, Anna Schiatti, Antonio Tempesta, Valeria Valdes			
LOMBARDIA	Ida Mangone, Veronica Vilei	ASST della Brianza	SSD di Malattie Endocrine, del Ricambio e della Nutrizione	Seregno (MB)
LOMBARDIA	Cesare Celeste Berra	IRCCS Multimedica	UO di Diabetologia e Malattie Metaboliche	Sesto San Giovanni (MI)
LOMBARDIA	Giancarla Meregalli, Denise Berzi, Danila Camozzi, Franco Forloni, Valentina De Mori, Lucia Valentini	ASST Bergamo Ovest	UOC Malattie Endocrine - Centro di riferimento regionale per il Diabete	Treviglio (BG)
LOMBARDIA	Stefano Fazion, Giovanni Rignanese, Marina Scaravelli, Gigliola Botta, Federica Chiari	ASST-Mantova, Ambulatorio di Viadana	SSD di Diabetologia e Malattie Metaboliche	Viadana (MN)
LOMBARDIA	Marta Di Stefano, Caterina Nucera, Maria Lauretta Anelli, Maria Grazia Tinelli, Ida Mangone	ASST della Brianza	SSD di Malattie Endocrine, del Ricambio e della Nutrizione	Vimercate (MB)
MARCHE	Valentino Cherubini, Valentina Tiberi, Antonio Iannilli, Monica Marino, Giada Boccolini, Antonia Capogna	Azienda Ospedaliero-Universitaria Ospedali Riuniti Ancona - Presidio G. Salesi	S.O.D. Diabetologia Pediatrica	Ancona (AN)
MARCHE	Francesca Silvetti , Lara Giovannini, Alessio Pieroni, Michele Perrone , Lucia Stella, Carlotta Peroni, Giorgia Squartini, Massimiliano Petrelli	Ospedali Riuniti delle Marche	Clinica di Endocrinologia e Malattie del Metabolismo	Ancona (AN)
MARCHE	Luigi Lanari, Federica Turchi, Maria Paola Luconi, Natascia Giorgini, Sonia Rosati, Magda Zenobi, Marco Federici, Beatrice Ciappini, Manuel Lagonigro, Elena Tortato	INRCA - IRCCS	UOC Malattie Metaboliche e Diabetologia	Ancona (AN)

REGIONE	AUTORI	OSPEDALE	REPARTO	CITTA'
MARCHE	Graziano Simonella, Marianna Galetta, Milena Santangelo, Federica D'Angelo, Rosa Anna Rabini	ASUR Marche AV5 - Ospedale Mazzoni	UOC Malattie Metaboliche e Diabetologia	Ascoli Piceno (AP)
MARCHE	Natalia Busciantella Ricci, Michelina Radatti	Area Vasta 3 Zona territoriale 10 Ospedale Civile Camerino	Diabetologia e Malattie Endocrino-metaboliche	Camerino (MC)
MARCHE	Cristian Quattrini, Clara Alessiani, Ornella Quarchioni	ASUR Area Vasta 3 - Distretto di Civitanova Marche	UOSD Diabetologia	Civitanova Marche (MC)
MARCHE	Vanessa Ronconi, Giorgio Montecchiani	AST Ancona-Ospedale E. Profili	UOSD Malattie Metaboliche e Diabetologia	Fabriano (AN)
MARCHE	Gabriella Garrapa, Anna Cani, Erica Landini, Giulio Lucarelli, Manuela Montoni, Isabella Occhialini, Lara Riccialdelli, Carla Spendolini	Ospedale Santa Croce-Azienda Sanitaria Territoriale di Pesaro Urbino	UOSD Endocrinologia e Diabetologia	Fano (PU)
MARCHE	Paola Pantanetti, Sandra Di Marco , Grazia Michetti, Giovanni Cangelosi, Cristina De Carolis, Marco Di Giacinti , Orietta Pazzi, Gianluca Cerasoli, Susanna Talevi, Sara Alberti	PO A. Murri - Asur Marche Area Vasta 4	UOSD Diabetologia	Fermo (FM)
MARCHE	Paola Canibus, Franco Gregorio, Francesca Paggi	Asur Marche - Area Vasta 2 - Jesi	UOSD malattie metaboliche e diabetologia	Jesi (AN)
MARCHE	Gabriele Brandoni, Francesca Carletti, Francesca Giampaoli, Manuela Ferroni, Anna Maria Frascati	Ospedale Santa Casa - ASUR Marche Area Vasta 2	Diabetologia	Loreto (AN)
MARCHE	Gabriele Brandoni, Brabara Polenta, Marilena Giovagnetti, Francesca Borroni , Andrea Benigni, Ilaria Peretti , Samuela Lardelli, Francesca Carletti, Vincenzo Carletti, Anna Maria	AV3 Presidio Ospedaliero Macerata	Diabetologia	Macerata (MC)

REGIONE	AUTORI	OSPEDALE	REPARTO	CITTA'
	Tesei, Jihane Lagtaa, Milena Corvini			
MARCHE	Luigi Maggiulli, Giulio Lucarelli, Alessandro Micillo, Anna Cani, Francesca Felicita, Giuseppina Fiori, Milena Giusti, Fabiola Lizzadro, Milena Paris, Antonio Russo, Antonella Sanchini	Ospedale San Salvatore - Azienda Sanitaria Territoriale Pesaro Urbino	UOSD Endocrinologia e Diabetologia	Pesaro (PU)
MARCHE	Gabriele Brandoni, Andrea Benigni, Ilaria Peretti , Maria Laura Scarpantoni, Rina Mozzoni, Francesca Carletti, Carla Oralndini, Tonia Lattanzi	Ospedale Santa Lucia - ASUR Marche Area vasta 3	Diabetologia	Recanati (MC)
MARCHE	Marianna Galetta, Graziano Simonella , Milena Santangelo, Federica D'Angelo, Rosa Anna Rabini	ASUR Marche AV5 - Ospedale Madonna Del Soccorso	UOC Malattie Metaboliche e Diabetologia	San Benedetto del Tronto (AP)
MARCHE	Silvana Manfrini, Silvia Rilli, Gessica Tinti, Daniela Pesce, Sebastiano Attardo	Asur Marche - Area Vasta 2 - Senigallia	U.O. Diabetologia	Senigallia (AN)
MARCHE	Maurizio Sudano, Claudio Molaioni, Carlucci Maria Assunta, Laura Biagiotti	Ospedale Urbino Asur Marche 1	SIT Diabetologia Endocrinologia	Urbino (PU)
MOLISE	Mariarosaria Cristofaro, Simonetta Di Vincenzo	A.S.R.e.M. Presidio Ospedaliero 'A.Cardarelli'	SC Endocrinologia - Diabetologia - Malattie Metaboliche	Campobasso (CB)
PIEMONTE	Emanuele Fraticelli, Elisabetta Benedusi, Andi Masha, Maria Elena Valera Mora, Teresa D'Ambrosio, Loredana Silvosi, Cristiana Olivero, Margherita Fissore, Manuela Morello, Giovanni La Motta	ASL CN02 Alba Bra	SSD Diabetologia e Malattie Metaboliche sede di ALba	Alba (CN)
PIEMONTE	Enrico Gabellieri, Paola Leporati, Alberto	AO SS Antonio e Biagio e Cesare Arrigo	SC Endocrinologia e Malattie Metaboliche 2	Alessandria (AL)

REGIONE	AUTORI	OSPEDALE	REPARTO	CITTA'
	Ragni, Emilia Biamonte, Giulia Bendotti, Marco Gallo		piano	
PIEMONTE	Chiara Bima, Piero Radaelli , Irene Samperi, Mara Steffanini, Isabella Bagatin, Monica Bertalli , Laura Cerini , Molinari Raffaella , Barbara Patrucco, Giovanna Ziero, Maria Chantal Ponziani	Ospedale SS Trinita' - ASl Novara	SSV Dipartimento di Malattie Metaboliche e Diabetologia	Borgomanero (NO)
PIEMONTE	Emanuele Fraticelli, Elisabetta Benedusi, Andi Masha, Maria Elena Valera Mora, Margherita Fissore, Teresa D'Ambrosio, Cristiana Olivero, Giovanni La Motta	ASL CN2 Alba Bra	SSD Diabetologia e Malattie Metaboliche- Sede Di Bra	Bra (CN)
PIEMONTE	Emanuele Fraticelli, Elisabetta Benedusi, Andi Masha, Maria Elena Valera Mora, Teresa D'Ambrosio, Loredana Silvosi, Cristiana Olivero, Margherita Fissore, Manuela Morello, Giovanni La Motta	ASL CN2 ALBA BRA	SSD Diabetologia e Malattie Metaboliche sede di Canale	Canale (CN)
PIEMONTE	Carlo Bruno Giorda, Giuliana Micali, Sandra Vendemiati, Liliana Audero, Antonela Crina Bursuc, Mirella Fornero, Loredana Cuccia, Francesco Romeo, Marella Doglio	Ospedale San Lorenzo - ASL TO5	S.C. Diabetologia Territoriale	Carmagnola (TO)
PIEMONTE	Carlo Bruno Giorda , Francesco Romeo, Origlia Carla, Elena Capello, Valentina D'Angelo, Tecla Marchese, Dolores Marzano, Loredana Cuccia, Anna Piovano, Cassandra Norma	Ospedale Maggiore di Chieri	S.C. Diabetologia Territoriale	Chieri (TO)

REGIONE	AUTORI	OSPEDALE	REPARTO	CITTA'
	Roma, Mariangela Incani, Simona Chiara, Patrizia Giai Via, Elisa Nada, Marisol Ibeth Cherigo , Barbara Tartaglino, Marella Doglio			
PIEMONTE	Riccardo Fornengo, Lidia Di Vito, Valentina Gatto, Stefano Allasia, Elisa Marinazzo, Maria Divina Pascuzzo	ASLTO4 Distretto di Chivasso e San Mauro e Distretto di Settimo Torinese	S.S.D. di Diabetologia	Chivasso (TO)
PIEMONTE	Francesco Caraffa, Daniela Gallo, Chiara Giulia Croce, Riccardo Fornengo	ASLTO4 Distretto di Ciriè	S.S.D. di Diabetologia	Cirie' (TO)
PIEMONTE	Francesco Tassone	AOS S.Croce e Carle Di Cuneo	Endocrinologia e Diabetologia	Cuneo (CN)
PIEMONTE	Giuseppe Placentino , Maura Rinaldi, Monica Adriana Resnik Scalella, Erika Mangione, Chiara Volpone, Danila Guida	Ospedale S. Biagio	SOC di Diabetologia e Malattie Metaboliche	Domodossola (VB)
PIEMONTE	Stefano Allasia, Elisa Marinazzo, Maria Divina Pascuzzo, Maria Pia Bavoso, Riccardo Fornengo, Chiara Giulia Croce, Piero Michele Modina	ASLTO4 Distretto di Ivrea e Distretto di Cuorgnè	S.S.D. di Diabetologia	Ivrea (TO)
PIEMONTE	Carlo Bruno Giorda, Roberta Manti, Giovanna Saraceno, Massimo Rainero, Cristina Linzalata, Cristina Laiolo, Rosa Mortello, Ornella Boscolo, Marella Doglio , Paola Chiaberti, Anna Paola Quaranta	Distretto Sanitario ASL TO5 sede di Moncalieri	S.C. Diabetologia Territoriale	Moncalieri (TO)
PIEMONTE	Carlo Bruno Giorda, Marella Doglio, Taisir Mahagna, Loredana Brocato, Giovanna Saraceno, Barbara Sieve, Dolores	Ospedale Maggiore - ASL TO5	Diabetologia e Malattie Metaboliche sede di Nichelino	Nichelino (TO)

REGIONE	AUTORI	OSPEDALE	REPARTO	CITTA'
	Marzano, Concetta Franchina, Filomena D'Angelo, Sandra Fasano, Simonetta Broggio			
PIEMONTE	Mauro Stroppiana, Silvia Maria Abate, Maria Elena Voglino, Marica Pinna, Rosetta Popolizio, Mirella Scarampi, Susanna Baldi, Marina Fresia, Lorella Tibaldi, Lorena Molino	ASL AT - Asti	Medicina Polifunzionale c/o Ospedale	Nizza Monferrato (AT)
PIEMONTE	Paolo Marzullo, Flavia Prodam, Marina Caputo, Gianluca Aimaretti, Tommaso Daffara, Valentina Bullara, Marco Zavattaro, Maria Grazia Mauri, Maria Teresa Samà	A.O.U. Maggiore della Carita' Novara	SCDU Endocrinologia	Novara (NO)
PIEMONTE	Giuseppe Placentino, Maura Rinaldi, Monica Adriana Resnik Scalella, Erika Mangione, Chiara Volpone	ASL Verbania - Cusio - Ossola (VCO) Piemonte	SSD Diabetologia e Malattie Metaboliche	Omegna (VB)
PIEMONTE	Enrico Pergolizzi, Anna Rosa Bogazzi, Giovanna Bendinelli	Ospedale di Pianezza ASL TO3	SSD Malattie Endocrine e Diabetologia	Pianezza (TO)
PIEMONTE	Enrico Pergolizzi, Paola Gennari, Daniela Gaia, Giuliana Micali, Patrizia Marino, Federica De Cicco, Katia Bonomo, Elisabetta Rossetto, Tiziana Stefani, Antonio Modarelli, Laura Bellino, Anna Clot	Ospedale Civile di Pinerolo ASL TO3	SSD Malattie Endocrine e Diabetologia	Pinerolo (TO)
PIEMONTE	Enrico Pergolizzi, Anna Rosa Bogazzi, Giovanna Bendinelli, Domenica Giuffrida, Marta Aventaggiato, Elisa Me	Ospedale di Rivoli ASL TO3	SSD Malattie Endocrine e Diabetologia	Rivoli (TO)

REGIONE	AUTORI	OSPEDALE	REPARTO	CITTA'
PIEMONTE	Enrico Pergolizzi, Maria Ausilia Caccavale, Mariantonietta Secchi	Ospedale Civile di Susa ASL TO3	SSD Malattie Endocrine e Diabetologia	Susa (TO)
PIEMONTE	Salvatore Oleandri, Elena Gramaglia, Enrico Mazza, Maria Antonietta Altea, Marcella Libera Balbo, Maria Pia Norma Bavoso, Alberto Blatto, Loredana Brocato, Francesco Brogna, Enrica Ciccarelli, Alessandra Clerico, Ginevra Corneli, Claudia De Natale , Fabiana Di Noi, Francesca Garino, Carlotta Gauna, Cristina Gottero, Piero Griseri, Michele Iaccarino, Alessandra Rita Mainolfi, Lisa Marafetti , Cristina Matteoda, Enzo Megale, Maria Divina Pascuzzo, Giuliana Petraroli, Valentina Ramella Gigliardi, Claudio Rossi, Silvia Rovere, Daniela Sansone, Claudia Toscano, Felicia Visconti	ASL Citta' di Torino - Ospedale Maria Vittoria	SC Endocrinologia Malattie Metaboliche	Torino (TO)
PIEMONTE	Maria Antonietta Altea , Marcella Libera Balbo, Maria Pia Norma Bavoso, Alberto Blatto, Loredana Brocato, Francesco Brogna, Enrica Ciccarelli, Alessandra Clerico , Ginevra Corneli, Claudia De Natale , Fabiana Di Noi , Francesca Garino, Carlotta Gauna , Cristina Gottero, Elena Gramaglia, Piero	ASL Citta' di Torino - Presidio Ospedaliero Oftalmico	SC Endocrinologia e Malattie Metaboliche	Torino (TO)

REGIONE	AUTORI	OSPEDALE	REPARTO	CITTA'
	Griseri, Michele Iaccarino, Alessandra Rita Mainolfi, Lisa Marafetti, Cristina Matteoda , Enrico Mazza, Enzo Megale, Salvatore Endrio Oleandri , Maria Divina Pascuzzo, Giuliana Petraroli, Valentina Ramella Gigliardi , Claudio Rossi, Silvia Rovere, Daniela Sansone , Maria Angela Seardo, Claudia Toscano, Felicia Visconti			
PIEMONTE	Maria Antonietta Altea, Marcella Libera Balbo, Maria Pia Norma Bavoso, Alberto Blatto, Loredana Brocato, Francesco Brogna, Enrica Ciccarelli , Alessandra Clerico, Ginevra Corneli, Claudia De Natale , Fabiana Di Noi , Francesca Garino, Carlotta Gauna, Cristina Gottero, Elena Gramaglia, Piero Griseri , Alessandra Rita Mainolfi, Lisa Marafetti, Cristina Matteoda, Enrico Mazza , Enzo Megale, Salvatore Oleandri , Maria Divina Pascuzzo , Giuliana Petraroli, Valentina Ramella Gigliardi, Claudio Rossi, Silvia Rovere , Daniela Sansone, Maria Angela Seardo , Claudia Toscano, Felicia Visconti	ASL Citta' di Torino - POLIAMBULATORIO C.so TOSCANA		Torino (TO)
PIEMONTE	Maria Antonietta Altea, Marcella Libera Balbo, Maria Pia Norma Bavoso,	ASL Citta' di Torino - LUNGO DORA SAVONA		Torino (TO)

REGIONE	AUTORI	OSPEDALE	REPARTO	CITTA'
	Alberto Blatto, Loredana Brocato, Francesco Brogna, Enrica Ciccarelli, Alessandra Clerico, Ginevra Corneli, Claudia De Natale, Fabiana Di Noi, Francesca Garino, Carlotta Gauna, Cristina Gottero, Elena Gramaglia, Piero Griseri , Michele Iaccarino, Alessandra Rita Mainolfi, Lisa Marafetti, Cristina Matteoda, Enrico Mazza , Enzo Megale, Salvatore Endrio Oleandri, Maria Divina Pascuzzo, Giuliana Petraroli, Valentina Ramella Gigliardi, Claudio Rossi , Silvia Rovere, Daniela Sansone , Maria Angela Seardo, Claudia Toscano, Felicia Visconti			
PIEMONTE	Maria Antonietta Altea , Marcella Libera Balbo, Maria Pia Norma Bavoso, Alerto Blatto, Loredana Brocato , Fracesco Brogna, Enrica Ciccarelli, Alessandra Clerico, Ginevra Corneli, Claudia De Natale, Fabiana Di Noi, Francesca Garino, Carlotta Gauna , Cristina Gottero, Elena Gramaglia, Piero Griseri, Michele Iaccarino, Alessandra Rita Mainolfi, Lisa Marafetti, Cristina Matteoda, Enrico Mazza , Enzo Megale, Salvatore Oleandri, Maria Divina	ASL Citta' di Torino - POLIAMBULATORIO MONTANARO		Torino (TO)

REGIONE	AUTORI	OSPEDALE	REPARTO	CITTA'
	Pascuzzo, Giuliana Petraroli, Valentina Ramella Gigliardi, Claudio Rossi, Silvia Rovere, Daniela Sansone, Maria Angela Seardo, Claudia Toscano, Felicia Visconti			
PIEMONTE	Lisa Marafetti, Cristina Matteoda, Enrico Mazza, Enzo Megale, Salvatore Endrio Oleandri, Maria Divina Pascuzzo , Giuliana Petraroli, Valentina Ramella Gigliardi, Claudio Rossi , Silvia Rovere, Daniela Sansone, Maria Angela Seardo, Claudia Toscano, Felicia Visconti, Maria Antonietta Altea, Marcella Libera Balbo, Maria Pia Norma Bavoso, Alberto Blatto, Loredana Brocato, Francesco Brogna, Enrica Ciccarelli, Alessandra Clerico, Ginevra Corneli, Claudia De Natale , Fabiana Di Noi , Francesca Garino, Carlotta Gauna, Cristina Gottero, Elena Gramaglia, Piero Griseri, Michele Iaccarino, Alessandra Rita Mainolfi , Lisa Marafetti	ASL Citta' di Torino - POLIAMBULATORIO PACCHIOTTI		Torino (TO)
PIEMONTE	Salvatore Oleandri	ASL Citta' di Torino - TORINO GORIZIA		Torino (TO)
PIEMONTE	Maria Antonietta Altea , Marcella Libera Balbo, Maria Pia Norma Bavoso , Alberto Blatto, Loredana Brocato,	ASL Citta' di Torino - TORINO MONGINEVRO		Torino (TO)

REGIONE	AUTORI	OSPEDALE	REPARTO	CITTA'
	Francesco Brogna, Enrica Ciccarelli, Alessandra Clerico, Ginevra Corneli, Claudia De Natale , Fabiana Di Noi , Francesca Garino, Carlotta Gauna, Cristina Gottero, Elena Gramaglia, Piero Griseri, Michele Iaccarino, Alessandra Rita Mainolfi, Lisa Marafetti, Cristina Matteoda , Enrico Mazza, Enzo Megale, Salvatore Endrio Oleandri, Maria Divina Pascuzzo, Giuliana Petraroli, Valentina Ramella Gigliardi, Claudio Rossi, Silvia Rovere, Daniela Sansone, Maria Angela Seardo, Claudia Toscano, Felicia Visconti			
PIEMONTE	Enrico Pergolizzi, Anna Rosa Bogazzi, Giovanna Bendinelli	Ospedale di Venaria Reale ASL TO3	SSD Malattie Endocrine e Diabetologia	Venaria Reale (TO)
PIEMONTE	Giuseppe Placentino, Maura Rinaldi, Monica Adriana Resnik Scalella, Erika Mangione, Chiara Volpone	Ospedale di Verbania	SOC di Diabetologia e Malattie Metaboliche	Verbania (VB)
PIEMONTE	Roberta Paltro, Simona Bandinelli, Luisella Savino, Arianna Busti, Valeria Cambria, Ilaria Leone	ASL VC	SS Diabetologia e Malattie Metaboliche	Vercelli (VC)
PUGLIA	Saverio Fatone, Alessandra Di Flaviani, Michelantonio Defano	ACISMOM Andria	Centro Diabetologico	Andria (BT)
PUGLIA	Stefania Annese, Matteo Paradiso, Chiara Corsano, Olga Lamacchia	OORR Riuniti di Foggia	Endocrinologia	Foggia (FG)

REGIONE	AUTORI	OSPEDALE	REPARTO	CITTA'
PUGLIA	Fabrizio Diacono	ASL Lecce - Poliambulatorio Martano	Ambulatorio di diabetologia	Martano (LE)
PUGLIA	Irene Alemanno, Lattanzio Cristiana	ASL Lecce DSS Poggiardo	Endocrinologia	Poggiardo (LE)
PUGLIA	Salvatore De Cosmo, Piscitelli Pamela , Rauseo Anna	IRCCS Casa Sollievo della Sofferenza	Medicina Interna - Endocrinologia	San Giovanni Rotondo (FG)
PUGLIA	Giuliana Cazzetta, Maria Teresa Branca, Marzia Filesi	ASL Lecce	Poliambulatorio Tricase	Tricase (LE)
PUGLIA	Giuliana Cazzetta, Claudia Toma	ASL Lecce	Poliambulatorio Distrettuale Ugento	Ugento (LE)
SARDEGNA	Giovanna Paola Tolu, Maria Pasqua Piras , Giuseppa Aguglia , Serafina Sini, Carla Verdi, Luisa Porcu	ASL Sassari , Distretto Sanitario di Alghero, Poliambulatorio	Servizio di Diabetologia e Malattie del Metabolismo, Distretto Sanitario di Alghero	Alghero (SS)
SARDEGNA	Efisio Cossu, Rosangela Maria Pilosu, Francesca Spanu, Maria Pina Puddu, Paola Pisanu, Antonietta Casu, Adelina Porru, Maria Vitalia Ortu , Maria Margherita Gessa, Carlotta Macis, Sabina Mereu , Giorgetta Farina	Presidio San Giovanni di Dio	Diabetologia e malattie metaboliche	Cagliari (CA)
SARDEGNA	Roberto Seguro, Francesca Scano, Valentina Maria Cambuli, Anna Rita Angioni	Ospedale San Michele Azienda Ospedaliera G. Brotzu	Struttura Complessa di Diabetologia	Cagliari (CA)
SARDEGNA	Alessandra Boi , Chiara Serafini, Sandro Cocco, Rossana Farci, Paola Elisa Meloni, Sergio Corsini, Lucia Milia, Assunta Poggi, Nada Richterova, Loredana Marongiu, Antonella Vanni , Giacomo Guaita	Presidio Ospedaliero Sirai -ASSL Carbonia - ATS Sardegna	Servizio di Diabetologia	Carbonia (SU)
SARDEGNA	Ilaria Pelligra, Giacomo Guaita ,	Ospedale Santa Barbara	Servizio Diabetologia Iglesias	Iglesias (SU)

REGIONE	AUTORI	OSPEDALE	REPARTO	CITTA'
	Cinzia Deias, Enzo Tuveri, Anna Maria Cardia , Fanny Marcello, Stefania Leoni, Graziella Angius, Emiliano Rosina, Gianfranco Orrù , Giuseppe Correale			
SARDEGNA	Maria Antonietta Fois, Laura Perra, Ada Pilia, Rosa Pilia, Giuliana Cao , Ada Pilia, Rosa Pilia, Giuliana Cao	ATS Sardegna ASSL Cagliari - Distretto di Sarcidano - Barbagia di Seulo e Trexenta	Servizio Territoriale Diabetologia di ISILI	Isili (CA)
SARDEGNA	Gisella Meloni, Maria Grazia Pani, Giacomo Marini, Claudia Putzu, Gilda Luisa Usala, Ornella Casula, Liliana Cerina, Andrea Cabiddu	ATS Sardegna - ASSL Lanusei	UO Diabetologia e Malattie Metaboliche	Lanusei (NU)
SARDEGNA	Luciana Maria Careddu, Giuseppina Gattu, Giacomo Marini , Paola Masala, Anna Franca Muroni, Alessia Prinzis, Liliana Sanna, Luisa Sollai, Paolo Giuseppe Michele Bianco	ASL Nuoro - Poliambulatorio Macomer	Ambulatorio di Diabetologia	Macomer (NU)
SARDEGNA	Efisio Cossu, Elena Loy, Marta Tuveri, Diego Mastino, Silvana Serra, Manuela Cauli, Giovanna Fabiana Zedda, Assunta Stella Belgiorno, Nisella Murtas	Azienda Ospedaliera Universitaria di Cagliari - Policlinico D. Casula	SSD Diabetologia	Monserrato (CA)
SARDEGNA	Alfonso Gigante, Serra Talia, Polo Rosalia , Bianco Paolo Giuseppe Michele , Cozzolino Sestilia, Sollai Luisa, Marteddu Liliana, Soro Alessandra, Ruiu Antonella, Pira Bastianina, Addis Alessandra, Litterio Lorella , Salaris	Ospedale C. Zonchello	Struttura Semplice Dipartimentale Malattie Metaboliche - Diabetologia	Nuoro (NU)

REGIONE	AUTORI	OSPEDALE	REPARTO	CITTA'
	Daniela, Isoni Serena, Cirillo Elisabetta			
SARDEGNA	Sara Cherchi , Maria Filippina Angius , Rosanna Vacca , Giulia Bacciu, Giancarlo Tonolo	ASL 2 Olbia - PO San Giovanni di Dio	S.C. Aziendale Diabetologia	Olbia (OT)
SARDEGNA	Concetta Clausi, Giuseppina Frau, Marco Mastinu, Luisa Sechi, Zoccheddu Simonetta, Melis Giuseppina	ASL Oristano - Ospedale San Martino	UO Malattie Metaboliche e Diabetologia	Oristano (SS)
SARDEGNA	Maria Antonietta Cambosu	ASL Nuoro	Polimabulatorio di Diabetologia- Distretto di Siniscola - Orosei	Orosei (NU)
SARDEGNA	Marco Mameli	DISTRETTO SANITARIO DI SASSARI - ASSL SASSARI - AST SARDEGNA	SERVIZIO DI DIABETOLOGIA - POLIAMBULATORI O DI PORTO TORRES	PORTO TORRES (SS)
SARDEGNA	Fernando Farci, Mariangela Ghiani, Rossella Cau, Maurizio Sparano, Danila Pistis, Anna Antonelli, Evelin Pandolfi, Valeria Tavolacci, Annalisa Cogoni, Maria Maddalena Pitturru	ATS Sardegna - ASSL Cagliari	UO Diabetologia Distretto di Quartu - Parteolla	Quartu Sant'Elena (CA)
SARDEGNA	Raffaella Derai, Marina Armeni, Chiara Satta, Cira Lombardo, Martina Melis, Stefania Casula, Vincenzo Sica, Maria Rosaria Meloni, Simonetta Mamusa, Riccarda Garau, Maria Antonietta Pisano, Elisabetta Cossu	ASL Sanluri Ospedale Nostra Signora di Bonaria	Diabetologia e Malattie Metaboliche	San Gavino Monreale (VS)
SARDEGNA	Alessandro Gentilini, Adolfo Pacifico, Pietro Fresu, Antonello Carboni , Tiziana Congiu, Margherita Idda, Angelo Fancellu	ATS Sardegna - ASSL1-Sassari-Distretto Di Sassari	Struttura Semplice di Diabetologia e Malattie Del Metabolismo	Sassari (SS)
SARDEGNA	Alessio Lai, Stefania	ATS Sardegna	Ambulatorio di	Senorbi (CA)

REGIONE	AUTORI	OSPEDALE	REPARTO	CITTA'
	Deiana, Tiziana Mudadu		Diabetologia - Poliambulatori di Senorbi	
SARDEGNA	Maria Antonietta Cambosu	Poliambulatorio di Siniscola ASL Nuoro	Poliambulatorio di Diabetologia- Distretto di Siniscola-Orosei	Siniscola (NU)
SARDEGNA	Jacopo Salvatore Bulla, Giuseppina Gattu	ASL 3 Nuoro - Distretto Sanitario Di Sorgono - Ospedale San Camillo	Servizio Diabetologia	Sorgono (NU)
SARDEGNA	Fabrizia Caucci	Distretto di Sassari	Polimabulatorio San Camillo - Diabetologia	Sorso - Sassari (SS)
SARDEGNA	Maria Chiara Cocco	Distretto Sanitario di Sarrabus Gerrei ASL 8 Cagliari	Ambulatorio Diabetologia Villasimius	Villasimius (CA)
SICILIA	Maurizio Di Mauro, Marco Di Mauro, Carmelo D'Urso, Maria Concetta Giuffrida	AOU Policlinico Vittorio Emanuele - P.O. Gaspare Rodolico	Centro diabetologico	Catania (CT)
SICILIA	Lucia Frittitta, Laura Sciacca , Agostino Milluzzo , Teresa Ballirò	ARNAS Garibarldi P.O. Garibaldi Nesima	UOSD Centro antidiabetico e per il trattamento dell'Obesità	Catania (CT)
SICILIA	Concetta Gatta, Caterina Merendino	A.Ospedaliero -Universitaria Policlinico San Marco	Divisione di Medicina Generale Presidio San Marco	Catania (CT)
SICILIA	Antonietta Maria Scarpitta, Antonino Lo Presti	P.O. Paolo Borsellino Marsala - A.S.P. Trapani	U.O.C Di Diabetologia e Malattie Metaboliche 7 Piano	Marsala (TP)
SICILIA	Giuseppina Russo, Annalisa Giandalia	A.O.U. Policlinico Universitario G. Martino	U.O.C. Medicina Interna - Malattie Metaboliche	Messina (ME)
SICILIA	Giuseppe Mattina	PTA Biondo	Ambulatorio di Diabetologia	PALERMO (PA)
SICILIA	Giuseppe Mattina, Giuseppe Mattina	PTA Palermo Centro	Poliambulatorio di Diabetologia	Palermo (PA)
SICILIA	Giovanni Saitta	Azienda Sanitaria Provinciale Di Messina	Poliambulatorio Distretto di Messina	Pistunina (ME)
SICILIA	Vittoria Sesta	ASP 8 Siracusa Ospedale Rizza	Poliambulatorio di Diabetologia	Siracusa (SR)
TOSCANA	Giovanna Gregori, Fabio Baccetti, Isabella Crisci, Mary Mori	Centro Polispecialistico Monterosso	SSD Diabetologia	Carrara (MS)
TOSCANA	Paola Orsini, Graziano	Ospedale Cecina	Diabetologia	Cecina (LI)

REGIONE	AUTORI	OSPEDALE	REPARTO	CITTA'
	Di Cianni			
TOSCANA	Loredana Rizzo, Giovanni De Gennaro, Walter Baronti, Laura Sambuco	Azienda USL Toscana Sud Est P.O. Misericordia	U.O.C. Diabetologia	Grosseto (GR)
TOSCANA	Stefania Bertoli, Margherita Occhipinti, Sabrina Cosimi	AUSL Toscana Nord Ovest - Ospedale Versilia	Reparto di Diabetologia	Lido di Camaiore (LU)
TOSCANA	Graziano Di Cianni, Paola Orsini, Graziano Di Cianni, Francesca Pancani, Emilia Lacaria, Giuseppe Viccica, Anna Turco	ASL Toscana Nord Ovest	U.O.C. Diabetologia	Livorno (LI)
TOSCANA	Alberto Di Carlo, Ilaria Casadidio, Ilaria Cuccuru, Cristina Lencioni	Ospedale Campo di Marte - AUSL Toscana Nord Ovest	Diabetologia e Malattie Metaboliche	Lucca (LU)
TOSCANA	Elisabetta Salutini, Claudia Cosentino, Anna Tedeschi, Roberto Anichini, Viti Secondina, Alice Valeria Magiar	Ospedale Cosma Damiano	UO Diabetologia Area Pistoiese	Pescia (PT)
TOSCANA	Graziano Di Cianni	Ospedale Villa Marina di Piombino - USL 6 Livorno	Sezione Diabetologia - U.O. Medicina Interna	Piombino (LI)
TOSCANA	Elisabetta Salutini, Claudia Cosentino, Anna Tedeschi, Roberto Anichini, Secondina Viti, Alice Valeria Magiar	Ospedale San Jacopo	UO Diabetologia Area Pistoiese	Pistoia (PT)
TOSCANA	Graziano Di Cianni, Paola Orsini, Laura Bini	Ospedale di Pontedera	Servizio di Diabetologia Pontedera	Pontedera (PI)
TOSCANA	Graziano Di Cianni, Paola Orsini	Ospedale di Portoferrario	Reparto di Diabetologia	Portoferraio (LI)
TOSCANA	Maria Calabrese, Caterina Lamanna, Marta Seghieri, Lucia Ianni, Monica Lorenzetti, Angela Marsocci, Sandra Guizzotti, Pamela Luccarini, Ylenia Vignoli, Graziella	Presidio Misericordia e Dolce - USL Toscana Centro	UOSD Diabetologia	Prato (PO)

REGIONE	AUTORI	OSPEDALE	REPARTO	CITTA'
	Scarcella, Carmelina Amendola			
TOSCANA	Graziano Di Cianni, Paola Orsini, Maria Norpoth	Ospedale di Volterra	Servizio di Diabetologia Volterra	Volterra (PI)
TRENTINO ALTO ADIGE	Bruno Fattor, Tiziano Monauni, Dalia Crazzolara, Ilaria Rubbo, Michela Cristini, Marco Dauriz, Roberta Endrighi	Ospedale Centrale di Bolzano	Servizio di Diabetologia	Bolzano (BZ)
TRENTINO ALTO ADIGE	Stefano Garavelli, Luisella Perina, Paola Marini, Sandro Inchiostro	APSS Ospedale S. Lorenzo di Borgo Valsugana	Centri Diabetologici di Borgo Valsugana, Pergine Valsugana e Primiero	Borgo Valsugana (TN)
TRENTINO ALTO ADIGE	Giuseppe Cheluci , Susi Martucci, Miriam Spadaro	Ospedale di Cavalese	UO Medicina	Cavalese (TN)
TRENTINO ALTO ADIGE	Florian Hermann Woehs, Giampiero Incelli, Christoph Pircher	Ospedale Tappeiner	Servizio Di Diabetologia	Merano (BZ)
TRENTINO ALTO ADIGE	Lorena De Moliner , Marlene Dall'Alda , Mariana Peroni, Katja Speese, Fabiola Frisinghelli, Ilaria Fait, Chiara Mazzucchi	Ospedale Santa Maria del Carmine di Rovereto	Ambulatorio di Diabetologia	Rovereto (TN)
TRENTINO ALTO ADIGE	Massimo Orrasch, Francesca Zambotti, Tiziano Lucianer, Bruna Barcatta, Silvia Clementi, Roberta Fellin, Cristina Faes, Ilaria Nicolao	APSS Trento Ospedale Santa Chiara Poliambulatorio Crosina	Centro Diabetologica	Trento (TN)
UMBRIA	Marisa Proietti	Azienda USL 2 Umbria	U.O. Diabetologia Amelia	Amelia (TR)
UMBRIA	Roberto Norgiolini, Viviana Minarelli, Antonio Mastroianni, Catiuscia Tamburi	Ospedale Civile Citta' di Castello	S.S. di Diabetologia	Citta' di Castello (PG)
UMBRIA	Maria Luisa Picchio, Chiara Pascucci, Francesca Cammilleri, Antonella Monni, Fabio Scarlato, Maria Cristina Celli	Centro di Salute Sede di Foligno ASL 2 dell' Umbria	Servizio di Diabetologia	Foligno (PG)

REGIONE	AUTORI	OSPEDALE	REPARTO	CITTA'
UMBRIA	Cecilia Marino, Silvia Arnone, Annarita Petrelli, Antonio Mastroianni	USL Umbria 1 Ospedale Branca di Gubbio	Servizio Diabetologia dell'Alto Chiascio	Gubbio (PG)
UMBRIA	Massimo Bracaccia, Elisabetta Mandosi, Marco Tonelli	Azienda USL Umbria 2	U.O. Diabetologia	Orvieto (TR)
UMBRIA	Elisabetta Torlone, Carmine Fanelli, Francesca Porcellati, Simone Pampanelli, Gabriele Perriello, Giuseppe Murdolo, Cristiana Vermigli, Nadia Biccari, Elisa Donnini, Valeriana Grassini, Maria Teresa Sulpizi, Francesca Mazzasette, Francesca Porcellati	Azienda Ospedaliera Santa Maria della Misericordia	SC Endocrinologia e Malattie del Metabolismo	Pereugia (PG)
UMBRIA	Paola Del Sindaco, Franca Ambrosi, Silvia Arnone, Luca Barbanera, Silvia Bellavita, Marinella Biagini, Sara Calandro, Roberta Celleno, Chiara Di Loreto, Ivonne Ghilardi, Debora Pezzuto, Laura Piastrella, Catiuscia Tamburi	USL 1 Umbria	UOS Diabetologia Distretto Del Perugino	Perugia (PG)
UMBRIA	Carlo Lalli, Francesca Cammilleri, Monia Cintio, Luca Sotgiu, Fabio Scarlato, Maura Scarponi	Distretto di Spoleto ASL 2 Umbria.	Servizio di diabetologia Poliambulatori Distrettuali	Spoleto (PG)
UMBRIA	Benedetta Carinella	Azienda USL Umbria 2	U.O. Diabetologia	Terni (TR)
VALLE D'AOSTA	Giulio Doveri, Emma Lillaz	Ospedale Regionale Umberto Parini	Ambulatori di Medicina-Diabetologia	Aosta (AO)
VENETO		Presidio Ospedaliero di Agordo	Medicina e Lungodegenza - Ambulatorio di Diabetologia Segreteria Medicina	Agordo (BL)
VENETO	Simonetta Lombardi, Daniele Raimondo,	Distretto Ovest (Arzignano, Valdagno,	UOSD di Diabetologia ed Endocrinologia	Arzignano (VI)

REGIONE	AUTORI	OSPEDALE	REPARTO	CITTA'
	Chiara Tommasi, Giovanni Romanello, Silvia Burlina, Dario Cioccoloni, Sabrina Cozza, Serena Sarti, Marta Binotto, Isabella Mecenero, Eliana Scott, Vilma Massariol, Alessia Fracca, Andrea Guiotto, Giovanna Tommasetto	Montecchio Maggiore, Lonigo)- ULSS 8 Berica	Territoriale	
VENETO	Alberto Marangoni, Sara Balzano, Rachele Scotton, Maria Ferrari, Alessandro Pianta	Ospedale Bassiano	Centro Antidiabetico	Bassano Del Grappa (VI)
VENETO	Corradina Alagona, Massimo Boaretto, Michela Da Rold, Claudia Cibien, Isabella Famà	ULSS 1 Dolomiti, Presidio Ospedaliero San Martino	UOS Diabetologia	Belluno (BL)
VENETO	Lucia Bondesan, Cristina Bittante	ASL 21 CAD Bovolone	Diabetologia	Bovolone (VR)
VENETO	Daniela Di Sarra	Poliambulatori Ospedale di Bussolengo - AULSS 9	UO di Medicina	Bussolengo (VR)
VENETO	Narciso Marin, Alessandra Cosma, Rachele Scotton, Sabrina Battagin, Antonella Maria Di Lucia , Catia Flori, Alessandro Bergamin, Moraika Riggi, Anna Pisacane, Agostino Paccagnella, Maria Lisa Marcon, Simone Girardin	Ospedale San Giacomo Apostolo - AULSS 2 Marca Trevigiana	UOS di Diabetologia	Castelfranco Veneto (TV)
VENETO	Andrea Nogara, Viola Sanga, Silvia Di Benedetto	Ospedale Civile di Chioggia ULSS 3 (ex ULSS 14)	UOSD Diabetologia e malattie del Ricambio	Chioggia (VE)
VENETO	Vera Frison, Anna Coracina, Michela Bettio, Alessio Filippi, Nino Cristiano Chilelli, Marco Grasso, Laura Tessarollo, Giuliana Taffarello, Felicia Abascia' , Sabrina	Presidio ospedaliero di cittadella, ulss 6 euganea	UOSD Diabetologia	Cittadella (PD)

REGIONE	AUTORI	OSPEDALE	REPARTO	CITTA'
	Cappellato, Emanuela Agostini, Silvia Convertini, Lisa Mason, Sandra Settimo, Isabella Zarantonello			
VENETO	Agostino Paccagella, Laura Nollino, Maria Sambataro, Sabina Marchetto , Maria Sara Persano, Barbara Almoto, Andrea Dotto, Teresa Benvenuti, Claudia Vigo, Marialisa Marcon, Annalisa Tittonel, Chiara Zanco, Federica Bianchin, Clementina Bravin, Federica Zambon, Sonia Sacco, Karen Brown	Presidio di Conegliano - USLS 7 Pieve di Soligo	UOSD Diabetologia	Conegliano (TV)
VENETO	Michele D'Ambrosio, Federica Tadiotto, Virgilio Da Tos	Ulss 6 Euganea Ospedale di Conselve	Centro U.O.S.D. Diabetologia	Conselve (PD)
VENETO	Maria Luisa Contin, Angela Pia De Cata, Isabella Negro, Iessica Iacovacci, Martina Colcera, Nicoletta Panzonato	Ospedale di Dolo	Servizio di Diabetologia	Dolo (VE)
VENETO	Ferruccio D'Incau, Anna Altomari, Antonio Volpi	Ospedale S. Maria del Prato - ULSS 2 - Feltre	UOS Diabetologia	Feltre (BL)
VENETO	Daniela Di Sarra	Poliambulatori Ospedale Isola - AULSS 9	CAD Ospedale Isola della Scala	Isola della Scala (VR)
VENETO	Carmela Vinci, Valeria Vallone	Ospedale di Jesolo - ASL n.10 Veneto Orientale	UOSD Diabetologia	Jesolo (VE)
VENETO	Lucia Bondesan, Cristina Bittante, Giulia Ceradini	ASL0 21 Verona CAD Legnago	Reparto di Medicina	Legnago (VR)
VENETO	Manuela Moise', Donata Barison	Distretto 2 ASL 3 Serenissima	Ambulatorio di DiabetologiaDistretto 2	Mestre (VE)
VENETO	Michele D'Ambrosio, Federica Tadiotto, Virgilio Da Tos,	Ulss 6 Euganea	U.O.S.D. diabetologia	Monselice (PD)

REGIONE	AUTORI	OSPEDALE	REPARTO	CITTA'
	Monica Saoncella			
VENETO	Michele D'Ambrosio, Federica Tadiotto, Virgilio Da Tos, Monica Saoncella	Ulss 6 Euganea Ospedale di Montagnana	Centro U.O.S.D. Diabetologia	Montagnana (PD)
VENETO	Narciso Marin, Alessandra Cosma, Rachele Scotton, Elisa Baldasso, Ivana Martini, Maria Zanatta, Lucia Favero, Espartaco Rigo, Maria Teresa Semenzin, Agostino Paccagnella, Maria Lisa Marcon, Simone Girardin	Presidio Ospedaliero di Montebelluna - AULSS 2 Marca Trevigiana	UOS di Diabetologia	Montebelluna (TV)
VENETO	Luciano Zenari, Lorenzo Bertolini, Francesca Grippaldi, Claudia Sorgato, Barbara Maistri	Ospedale Sacro Cuore Don. Calabria	U.O. di Diabetologia (6 piano)	Negrar (VR)
VENETO	Maria Luisa Contin , Michela Dal Pos, Martina Aldrigo, Barbara Centenaro, Loris Bortolato, Nicoletta Bussola	USL 13 di Mirano Ospedale di Noale	Servizio di Diabetologia di Noale	Noale (VE)
VENETO	Lucia Bondesan, Marques Alessandra	ASL 21 CAD Nogara	Reparto di Medicina	Nogara (VR)
VENETO	Maria Simoncini, Marco Strazzabosco	Ospedale Milani	Ambulatorio Diabetologico	Noventa Vicentina (VI)
VENETO	Gian Paolo Fadini, Alberto Maran, Saula Vigili De Kreutzenberg, Benedetta Maria Bonora, Mario Luca Morieri, Nicola Vitturi, Cristina Crepaldi, Monica Vedovato, Daniela Bruttomesso, Federico Boscari, Mauro Rigato, Andrea Bruttocao, Gabriella Guarneri, Angelo Avogaro	Azienda Ospedaliera di Padova	Dipartimento di Medicina - UOC Malattie del Metabolismo	Padova (PD)
VENETO	Annunziata Lapolla, Francesco Piarulli,	Universita' degli Studi di Padova - UOC	UOC di Diabetologia e Dietetica	Padova (PD)

REGIONE	AUTORI	OSPEDALE	REPARTO	CITTA'
	Giovanni Sartore, Giuseppe Bax , Maria Grazia Dalfrà, Michela Masin , Silvia Minardi, Alessandra Gallo , Barbara Bonsembiante , Isabella Negro, Silvia Longhin	Diabetologia e Dietetica USL 16 Padova		
VENETO		Ospedale di Pieve di Cadore	UO di Medicina - Servizio di Diabetologia	Pieve di Cadore (BL)
VENETO	Cristiano Fongher	Casa di Cura Madonna della Salute - ULSS 5	Ambulatorio di Diabetologia	Porto Viro (RO)
VENETO	Carmela Vinci, Isabella Massimina Colletti, Milena Sira Zanon, Michela Signorato, Anna Del Bianco, Antonella Garro	ASL 10 Veneto Orientale Ospedale di Portogruaro	UOSD Diabetologia	Portogruaro (VE)
VENETO	Carmela Vinci, Valeria Vallone, Jessica Cristaldi, Antonio De Mitri, Cristina Toffolo	Ospedale S. Dona Di Piave ASL 10 Veneto Orientale	Servizio di Diabetologia	San. Donà Di Piave (VE)
VENETO	Silvana Costa, Marco Strazzabosco	Ospedale Territoriale di Sandrigo	Ambulatorio Diabetologico	Sandrigo (VI)
VENETO	Francesco Calcaterra, Antonella Senesi, Marina Miola, Francesca Dal Molin, Giulia Faccin, Chiara Trappolin	Casa della Salute di Schio	U.O. Diabetologia Endocrinologia Dietetica	Schio (VI)
VENETO	Lucia Gottardo, Valentina Mariano	AULSS 3 Serenissima - Ospedale SS Giovanni e Paolo	UOSD Ipertensione e Patologie Endocrino Metaboliche e Angiologiche	Venezia (VE)
VENETO	Elisabetta Brun, Maria Simoncini, Chiara Alberta Mesturino, Consuelo Grigoletto, Silvana Costa, Marco Strazzabosco	Ospedale San Bortolo -ULSS 6 Vicenza	U.O. Endocrinologia e Malattie del Metabolismo	Vicenza (VI)
VENETO	Daniela Di Sarra	CAD Villafranca - Poliambulatori Ospedale Villafranca - AULSS 9	CAD Villa Franca	Villafranca Veronese (VR)
VENETO	Lucia Bondesan	ASL 21 CAD Zevio	Centro Antidiabetico	Zevio (VR)
